# Murine and Humanized Mouse Models in Autoimmune Disease Research and Therapeutics Development

**DOI:** 10.3390/biology15141125

**Published:** 2026-07-10

**Authors:** Sameena Nikhat, Suman Bose, Mohsen Khosravi-Maharlooei

**Affiliations:** 1 Department of Immunology, Mayo Clinic, Phoenix, AZ 85054, USA; nikhat.sameena@mayo.edu (S.N.); bose.suman@mayo.edu (S.B.); 2 Department of Physiology and Biomedical Engineering, Mayo Clinic, Phoenix, AZ 85054, USA; 3 Department of Biochemistry and Molecular Biology, Mayo Clinic, Phoenix, AZ 85054, USA

**Keywords:** autoimmune disease, animal models, humanized mice, inflammatory bowel disease, multiple sclerosis, type-1 diabetes, rheumatoid arthritis, immune tolerance, adaptive immunity, human immune system (HIS)

## Abstract

Autoimmune diseases arise from a breakdown of immune tolerance and complex interplay of genetic susceptibility, environmental triggers, tissue-specific immune responses, microbiota, and regulatory pathways. Mouse models remain essential for dissecting these mechanisms, but no single model fully reproduces the heterogeneity, chronicity, and immune complexity of autoimmune disease in humans. This review summarizes classical murine and humanized mouse models used to study inflammatory bowel disease (IBD), multiple sclerosis (MS), type-1 diabetes (T1D), and rheumatoid arthritis (RA). We also highlight disease-specific scoring systems, including clinical indices, histopathology, imaging, cytokine profiling, autoantibody assessment, and human immune-cell readouts, as essential tools for standardized interpretation. Conventional murine models provide experimental control and mechanistic clarity, whereas humanized models improve assessment of human immune responses, patient-specific biology, and therapeutic translation. However, humanized systems remain limited by incomplete immune reconstitution, graft-versus-host disease, donor variability, cost, and incomplete tissue architecture. By providing a comparative framework spanning both conventional and humanized models, this review aims to guide informed model selection tailored to specific research questions in autoimmune disease biology and translational therapeutic development.

## Simple Summary

Autoimmune diseases occur when the immune system mistakenly attacks the body’s own tissues. These diseases are difficult to study directly in humans because early disease events are often inaccessible, and disease mechanisms vary between patients and organs. Mouse models have therefore been essential for understanding how immune tolerance fails, how inflammation damages tissues, and how new therapies may work. However, no single mouse model fully reproduces human autoimmune disease. Some models are best for studying defined immune pathways or tissue injury, whereas others better capture chronic disease progression or human immune responses. This review summarizes murine and humanized mouse models used to study inflammatory bowel disease, multiple sclerosis, type-1 diabetes (T1D), and rheumatoid arthritis. We compare their disease features, applications, advantages, limitations, and commonly used readouts. We also highlight the importance of choosing models based on specific research questions such as disease initiation, immune mechanisms, tissue damage, biomarker discovery, or therapeutic testing. Overall, this review provides a practical framework to help researchers select and interpret animal models more carefully in autoimmune disease research.

## 1. Introduction

Autoimmune diseases comprise a diverse group of chronic inflammatory disorders caused by a failure of immunological tolerance, leading to sustained immune-mediated damage of self-tissues [1,2]. These diseases can be broadly classified as organ-specific or systemic and collectively represent a major global health burden [3,4,5]. Despite differences in clinical presentation and target organs, autoimmune diseases share common pathogenic principles rooted in dysregulated immune recognition, aberrant lymphocyte activation, and defective regulatory mechanisms that normally maintain self-tolerance [1,3,6,7]. Regulatory T cells (Tregs) are central to maintaining peripheral tolerance [8], yet increasing evidence indicates that autoimmunity is not simply associated with numerical Treg deficiency. Instead, Tregs often accumulate at sites of inflammation but exhibit impaired suppressive function, altered metabolic states, or inflammatory reprogramming toward effector-like phenotypes [9,10,11].

In addition, genetic susceptibility strongly influences autoimmune risk, with the major histocompatibility complex (MHC) representing the most significant contributor across diseases [3,12]. At the cellular and molecular level, autoimmune pathology reflects an imbalance between pro-inflammatory effector programs and immunoregulatory pathways. Tissue microenvironments further shape autoimmune responses by integrating immune, stromal, epithelial, and microbial signals. Therefore, disruption of epithelial integrity, altered microbial composition, or dysregulated stromal signaling results in alteration of regulatory responses [13,14].

Genome-wide association studies (GWAS) have identified hundreds of non-MHC risk loci implicating pathways involved in antigen presentation, T-cell receptor activation and signaling, cytokine responsiveness, and lymphocyte differentiation, including IL-2/STAT5, IL-23/Th17, and costimulatory signaling networks [3,15,16,17]. It should be emphasized, however, that GWAS identify disease-risk loci rather than causal genes: each associated locus typically encompasses many polymorphisms and multiple candidate genes, and even the strongest associations (e.g., the HLA region in MS, RA, and T1D) have not been conclusively resolved to the exact causative variants or their functions. The candidate genes implicated by GWAS should therefore be regarded as provisional rather than established. Notably, genetic dissection of complex autoimmune disease in mouse and rat models has, in several instances, been more productive than human GWAS for positioning and functionally validating causal variants. A paradigmatic example is *Ncf1*, which encodes the p47phox subunit of the NOX2 NADPH oxidase complex required for reactive oxygen species (ROS) production during immune oxidative burst. In rat arthritis models, positional cloning of pristane-induced arthritis quantitative-trait loci (PIA QTL) identified *Ncf1* as a regulator of arthritis severity, with disease-associated variants showing decreased oxidative burst but enhanced autoreactive T-cell responses [18]. This mechanism was then functionally confirmed in mouse models of arthritis and encephalomyelitis. Subsequent targeted human genetic studies showed that the NCF1 p.Arg90His variant and NCF1 copy-number variation are associated with susceptibility to systemic lupus erythematosus (SLE), Sjögren’s syndrome, and RA, explaining why this locus was difficult to resolve by conventional GWAS [19]. Therefore, animal models can help move from broad disease-associated loci to functionally validated causal risk genes.

## 2. Animal Models for Studying Autoimmune Disorders

Animal models have been indispensable for elucidating the mechanisms governing immune tolerance and autoimmunity. Spontaneous, genetically engineered, induced, and humanized mouse models have provided fundamental insights into lymphocyte selection, the balance between effector and regulatory T cells, and tissue-specific immune regulation. However, each model reproduces only selected aspects of human disease, necessitating careful interpretation and the complementary use of multiple models (wherever appropriate).

In this review, we examine classical and emerging murine and human immune system (HIS) mouse models of IBD, MS, T1D, and RA. For each model, we describe its disease manifestations, course and time frame, susceptible genetic background, phenotype and pathology, applications, advantages, and inherent limitations. These features facilitate comparison and selection of an appropriate model for the research question being addressed. For each disease, we also describe relevant clinical, histopathological, and radiological scoring approaches and expected outcomes to support standardized assessment of disease severity, progression, and therapeutic response.

Major biological features of autoimmunity can be mapped to the experimental systems best suited to investigate them. Genetic susceptibility is commonly examined using spontaneous, congenic, and HLA-transgenic or knock-in models; tissue-resident and barrier-dependent immunity requires models that preserve the relevant epithelial, stromal, endothelial, or glial niches; and microbiota-driven mechanisms require microbiota-controlled or gnotobiotic systems. TCR-transgenic and adoptive-transfer models are particularly useful for dissecting antigen-specific tolerance and its breakdown, whereas humanized and HLA-restricted models can improve the assessment of human-specific immune mechanisms and therapeutics. Therefore, model selection should integrate the relevant disease driver, specific research question, and expected outcome.

### 2.1. Inflammatory Bowel Disease

IBD, encompassing Crohn’s disease (CD) and ulcerative colitis (UC), is a chronic inflammatory disorder of the gastrointestinal tract characterized by disrupted mucosal homeostasis, aberrant immune activation, epithelial barrier dysfunction, and dysregulated host–microbiome interactions. UC is restricted to the colon and is characterized by superficial inflammation (restricted to mucosa and sub-mucosa) and rectal bleeding [20]. On the other hand, CD affects the entire intestine with inflammation penetrating transmurally, causing perforation, strictures, and extensive fibrosis (requiring surgery in up to 80% of patients). The etiology of IBD is multifactorial, involving complex interactions between genetic susceptibility, environmental triggers, gut microbiota, epithelial cells, innate immune cells, APCs, T cells, B cells, stromal cells, cytokines, and chemokines [21,22]. Genome-wide association studies have identified over 200 susceptibility loci, including variants in NOD2, ATG16L1, IRGM, IL23R, CARD9, JAK2, STAT3, and genes involved in microbial sensing, autophagy, epithelial defense, cytokine signaling, and lymphocyte differentiation [22,23]. Environmental factors such as diet, smoking (protective in UC but detrimental in CD), antibiotic exposure, and infections further modulate disease risk and progression [2,4].

CD is commonly associated with Th1/Th17-skewed inflammation involving IFN-γ, IL-12/IL-23, IL-17, IL-22, and TNF-α pathways, with GM-CSF and myeloid-cell programs increasingly recognized as additional context-dependent contributors [24,25,26]. In contrast, UC has been linked to an atypical Th2-like response involving non-classical CD1d-restricted NKT cells and IL-13-mediated epithelial barrier injury, although both diseases show overlapping and heterogeneous cytokine networks [27,28,29]. Environmental factors, including diet, smoking, antibiotic exposure, infections, medication exposure, stress, and early-life microbial perturbations, further shape disease risk and progression [30].

Table 1 describes the murine and humanized models for studying IBD. The selection of a given IBD model is dependent on the dominant disease driver. For example, Dextran Sulfate Sodium (DSS) is most appropriate for epithelial injury, barrier dysfunction, and innate immune activation [31,32,33,34,35,36,37], whereas the 2,4,6-trinitrobenzene sulfonic acid (TNBS) [38,39,40,41] and oxazolone [42,43] models are more useful for T-cell-mediated and cytokine-skewed mucosal inflammation. Genetically driven models such as *Muc2*^-/-^ [44,45,46,47] and *C3gnt*^−/−^ mice [48,49,50] model mucus-barrier defects, while NEMO^IEC-KO^ mice [51,52,53,54] reproduce epithelial-cell death, barrier breakdown, and microbiota-driven spontaneous colitis. *Il-10*^-/-^ [55,56,57,58], *Stat3*^-/-^ [59,60], and TRUC [61,62] models provide platforms for studying chronic host–microbiota interactions and defective immune regulation. The SAMP1/YitFc model [63,64] develops spontaneous Crohn’s disease-like ileitis and is useful for studying leukocyte trafficking, Th1/TNF-driven inflammation, fibrosis, and chronicity. Finally, the CD45RB^hi^CD4^+^ T-cell transfer model [65,66,67,68] enables investigation of T-cell-intrinsic mechanisms and Treg-mediated suppression in microbiota-dependent colitis.

Humanized IBD models add translational value by enabling assessment of patient-derived immune responses, human cytokine profiles, Treg-targeted therapies, and human Treg pathways. These include UC-PBMC-humanized [69,70,71,72] and CD-PBMC-humanized [72,73] NSG models, CD34^+^ hematopoietic stem cells (HSCs)-humanized TNBS colitis models treated with low-dose IL-2 [74], PBMC-humanized DSS colitis models for autologous Treg therapy [75], and CD4^+^ T-cell-humanized NSG-Aβ°DR1 models for testing AHR-mediated Treg induction [76]. However, these systems remain limited by incomplete human intestinal epithelial, stromal, vascular, microbial, and myeloid compartments (see Section 5 for further details). Thus, IBD studies are strongest when acute injury models, spontaneous ileitis models, genetic/barrier models, transfer systems, and humanized platforms are used complementarily to separate epithelial damage, immune-mediated disease progression, and human translational responses.

Accurate and reproducible assessment of disease severity in experimental colitis models requires integration of clinical, macroscopic, and histopathologic parameters. The most used clinical metric is the Disease Activity Index (DAI), which combines quantitative measurements of body weight loss (percentage loss relative to baseline), stool consistency (graded from well-formed to liquid diarrhea), and presence of fecal blood (occult or gross) into a composite score assessed daily during disease induction [77,78]. At endpoint, macroscopic indicators such as colon length shortening, wall thickening, and splenomegaly provide additional indices of inflammation severity. Histopathologic evaluation is performed on formalin-fixed, paraffin-embedded colon sections stained with hematoxylin and eosin (H&E). Scoring systems typically quantify epithelial damage (crypt loss, ulceration, goblet cell depletion), inflammatory cell infiltration, and extent of lesion distribution (mucosal vs. transmural involvement) [79]. Semi-quantitative scales (usually 0–3 or 0–4 per parameter) are summed to generate a total histologic score, and blinded scoring by independent observers is strongly recommended to reduce bias. In chronic models, additional parameters such as crypt hyperplasia, architectural distortion, and fibrosis are included [68]. For mechanistic studies, clinical scoring must be complemented by quantitative measures for cytokine profiling (TNF-α, IL-6, IL-17A, IFN-γ), and flow cytometric analysis of lamina propria immune subsets to correlate disease severity with underlying immunopathology [35,80,81].

### 2.2. Multiple Sclerosis

MS is a chronic inflammatory demyelinating disease of the central nervous system (CNS) characterized by focal immune cell infiltration, myelin destruction, axonal injury, and progressive neurodegeneration [82]. Genetic susceptibility is strongly linked to the MHC class-II allele HLA-DRB1*15:01, with numerous other loci implicated in antigen presentation and cytokine signaling pathways, and vitamin D metabolism [83,84,85], while Epstein–Barr virus (EBV) infected B cells significantly increase disease risk [86,87]. Pathogenesis is driven by autoreactive CD4^+^ T cells recognizing myelin antigens (myelin basic protein (MBP), myelin oligodendrocyte glycoprotein (MOG) and proteolipid protein (PLP)) that become activated in the periphery and differentiate into pro-inflammatory Th1 and Th17 subsets [88]. Although early studies emphasized IFN-γ–producing Th1 cells, subsequent work showed IL-23-dependent Th17 cells and GM-CSF-producing T cells mediate disruption of the blood–brain barrier and activate resident microglia and macrophages, thus causing CNS inflammation [89,90,91]. Cytotoxic CD8^+^ T cells further mediate axonal injury while B cells further contribute to intrathecal antibody production and antigen presentation [92,93]. Moreover, foundational work demonstrated that Tregs from MS patients have been reported to exhibit impaired suppressive function [94].

In Table 2, we discuss classical EAE as well as other murine and humanized models for MS. Animal models for MS differ most clearly in whether they model autoimmune initiation, effector-cell trafficking, demyelination, or repair. Active EAE remains the most widely used platform for studying antigen-driven CNS autoimmunity, Th1/Th17 responses, immune trafficking, and neuroinflammation [95,96,97,98]. Passive EAE provides a more reductionist system for testing encephalitogenic T-cell function and effector mechanisms, but it bypasses the priming phase of disease [99,100,101,102]. Cuprizone-induced demyelination is highly useful for studying oligodendrocyte loss, remyelination, and glial responses, yet it is not a true autoimmune model because adaptive immunity is not required [103,104,105]. Viral models such as Theiler’s murine encephalomyelitis virus (TMEV) add value for studying infection-triggered neuroinflammation and chronic demyelination [106,107,108,109]. Humanized and HLA-transgenic models are particularly relevant for questions involving human antigen presentation, EBV-associated mechanisms, or human immune-cell contributions [87,110,111,112,113,114,115]. Therefore, model selection for MS should be guided by the specific process under investigation: immune priming, CNS immune-cell trafficking, demyelination, remyelination, viral-triggered neuroinflammation, or human antigen-restricted immune responses. Since each model interrogates fundamentally different processes, therapeutic efficacy in one model frequently does not predict efficacy in another. For example, agents that suppress immune priming or CNS trafficking in EAE may have no effect on the oligodendrocyte injury and remyelination measured in the cuprizone model, and vice versa, so the endpoint (immune suppression versus myelin repair) must match the chosen model.

EAE disease severity is commonly assessed using clinical scoring systems to assess neurological deficits in a reproducible and semi-quantitative manner. Mice are examined daily following immunization, and disease activity is graded using a 0–5 scale based on ascending motor impairment (0, no clinical signs; 1, limp tail or loss of tail tone; 2, hind limb weakness or abnormal gait; 3, partial hind limb paralysis; 4, complete hind limb paralysis; and 5, moribund state or death) [95,116]. Additionally, body weight monitoring is routinely performed as a supportive disease index. Histopathologic evaluation complements clinical scoring and includes quantification of inflammatory cell infiltrates, demyelination (commonly assessed by Luxol Fast Blue staining), axonal injury, and gliosis within the spinal cord and brain [95,116]. In mechanistic studies, flow cytometric analysis of CNS-infiltrating immune cells and cytokine profiling (e.g., IFN-γ, IL-17A, GM-CSF) are incorporated to correlate clinical severity with underlying immunopathology [111].

**Table 2:** Most common animal models for Multiple Sclerosis.

Model	Description	Phenotype and Pathology	Applications	Advantages	Limitations	References
***Murine Models***						
Experimental autoimmune encephalomyelitis (EAE) mice	C57BL/6 (B6), SJL/J, PL/J, or NOD mice immunized with myelin antigens (MBP, MOG and PLP). MOG_35–55_ peptide induces a chronic disease while PLP_139–151_ induces a relapsing-remitting disease course. **Disease course and time frame:** Acute monophasic or chronic/relapsing depending on strain and antigen. Onset ~9–14 days post-immunization; peak ~ 18–25 days. **Genetic background:** C57BL/6 (MOG_35–55_, chronic) and SJL/J (PLP_139–151_), relapsing-remitting.	‘Classical EAE’ involving auto-reactive Th1/Th17 CD4^+^ T cells infiltrating CNS causing demyelination (peaks 1–2 weeks post-injection, primarily confined to the spinal cord); ascending flaccid paralysis and axonal damage; involves both resident microglia and infiltrating monocyte-derived macrophages; increased expression of IFN-γ and IL-17. ‘Atypical EAE’ also involves brain inflammation.	Autoimmune-mediated inflammation, demyelination and axonal damage in the CNS; cytokine networks; immune trafficking; tolerance mechanisms and disease-modifying triggers (DMTs); PLP-induced EAE allows studying of relapsing-remitting MS.	Gold standard—recapitulates key features of MS including inflammatory infiltration of the CNS, demyelination, axonal damage, and neurological dysfunction.	Strain-specific disease severity; lesions primarily confined to spinal cord (no brain involved) in most models; synthetic peptides lack post-translational modifications seen on endogenous myelin.	[95,96,97,98]
Adoptive transfer EAE (Passive EAE)	Transfer of activated myelin-specific CD4^+^ Th1 or Th17 cells from immunized donors into naïve syngeneic recipients, bypassing priming phase. **Disease course and time frame:** Acute/passive EAE; onset ~4–10 days after cell transfer, peak ~10–15 days. **Genetic background:** Syngeneic recipients; commonly C57BL/6 for MOG-specific cells, SJL/J for PLP-specific cells, and B10.PL/PL/J for MBP-specific cells.	Rapid onset CNS inflammation, paralysis, T-cell infiltration, demyelination; Th1-polarized myelin-specific CD4^+^ T cells produce IFN-γ, TNF-α, and GM-CSF while IL-23/Th17-polarized cells produce IL-17A, IL-22 and GM-CSF.	Study effector phase of EAE, T-cell pathogenicity; CNS infiltration, cytokine function; track encephalitogenic T cells in vivo.	Highly controlled; isolates T-cell-driven pathology; useful in characterizing T-cell effector function in the CNS.	Does not model disease initiation; reduced complexity.	[99,100,101,102]
Cuprizone model	Oral administration of cuprizone (a copper chelator) induces selective oligodendrocyte apoptosis (via mitochondrial dysfunction) and reversible demyelination. **Disease course and time frame:** Demyelination ~3–6 weeks of cuprizone feeding; remyelination after withdrawal. **Genetic background:** Young adult C57BL/6 mice; response varies with strain, age, and sex.	Toxin-induced robust demyelination, oligodendrocyte depletion, microglial activation, and astrocytosis without T- and B-cell involvement; toxin withdrawal allows remyelination driven by oligodendrocyte progenitors.	Studying oligodendrocyte biology, myelin degeneration, remyelination mechanisms, glial responses.	Highly reproducible; allows precise temporal control of demyelination/remyelination; avoids confounding adaptive immune responses; ideal for repair and regeneration studies.	Does not model immune initiation of MS; minimal overt neurological deficits despite significant pathology; strain, age, and sex-dependent variability; highly restricted remyelination upon prolonged exposure.	[103,104,105]
Theiler’s Murine Encephalomyelitis Virus (TMEV) model	Intracerebral infection with Theiler’s murine encephalomyelitis virus (TMEV); biphasic disease -early acute encephalitic phase followed by persistent viral infection and chronic immune-mediated demyelination. **Disease course and time frame:** Biphasic viral disease; acute encephalitic phase ~1–2 weeks; chronic demyelination begins ~1-month post-infection and may persist long-term. **Genetic background:** SJL/J mice are susceptible; C57BL/6 is relatively resistant.	Virus-induced activation of macrophages, microglia and B cells; T-cell infiltration; chronic progressive CNS demyelinating disease with persistent spinal cord inflammation, demyelinated plaques, axonal injury, and progressive motor deficits; failure to clear TMEV leads to chronic demyelination; viral infectivity of APCs determines strain-specific susceptibility.	Studying virus-triggered demyelination, progressive MS-like pathology, epitope spreading, molecular mimicry/bystander activation, and neurodegeneration; therapies targeting progressive demyelination.	More relevant than toxin-induced models for studying immune-mediated chronic demyelination; captures features not well modeled by acute EAE, including viral trigger and CNS viral persistence.	Viral etiology does not represent all MS cases; disease is highly mouse and virus-strain dependent.	[106,107,108,109]
TCR-Transgenic (Tg) EAE mice	Genetically engineered for myelin antigen-specific CD4^+^ T cells expressing a MOG-specific TCR (2D2) or MBP-specific TCR (Tg4). **Disease course and time frame:** Spontaneous or accelerated EAE; variable onset from weeks to months, or faster after immunization/activation. **Genetic background:** 2D2 MOG-specific TCR mice on C57BL/6 and Tg4 MBP-specific TCR mice commonly on B10.PL backgrounds.	Spontaneous autoimmunity or accelerated EAE upon minimal stimulation; presence of autoreactive T cells in peripheral repertoire; increased Th1/Th17 differentiation under inflammatory conditions.	Studying tolerance breakdown; defined TCR specificity allows precise Th1/Th17 mechanistic dissection.	Allows insights on initial mechanistic events in pathogenesis; ideal for Treg and checkpoint studies.	High frequency of artificial autoreactive T cells does not mimic polyclonality in MS; low and variable disease incidence, often requires triggers (e.g., adjuvants, infections).	[117,118]
***Humanized mouse models***						
HLA-Tg and TCR/HLA double-Tg EAE mice	MHC-KO mice expressing MS-associated HLA haplotypes (e.g., HLA-A2 variants) alone or in combination with myelin-reactive human. **Disease course and time frame:** Induced or spontaneous disease (onset ~2–3 weeks after immunization), or spontaneous disease over months in selected double-Tg models. **Genetic background:** MHC^-/-^ C57BL/6 expressing MS-associated HLA class-II alleles.	HLA-restricted antigen presentation; disease induction with human myelin antigens or autoreactive T cells; CD4^+^ Th1/Th17-driven pathology; demyelination and paralysis.	Studying HLA-dependent antigen recognition and TCR specificity; epitope mapping of MS-relevant peptides; antigen-specific tolerance regulation.	Directly models human genetic susceptibility and TCR autoreactivity; highly reproducible.	Immune system remains murine in single Tg mice; Double Tg mice do not fully model human T-cell repertoire in MS; limited B-cell and antibody relevance.	[110,111,112,113]
Humanized PBMC *B2m*-NOG mice	*B2m*-NOG mice transplanted with human PBMCs from HLA-DRB1-genotyped MS patients. **Disease course and time frame:** Spontaneous or induced human T-cell CNS lesions within weeks after PBMC engraftment. **Genetic background:** *B2m*-NOG mice (NOD.Cg-*B2m^-/-^Prkdc(scid) Il2rg(tm1Sug)*/JicTac).	Spontaneous human CD8^+^ T-cell lesions in non-immunized mice, mixed CD8/CD4 T-cell lesions in EAE-immunized mice; brain and spinal cord involvement.	Study donor genetics in disease pathology to test donor-stratified therapies.	CD8^+^ T-cell lesions in the brain and spinal cord and B-cell engraftment closely recapitulate MS pathology.	Limited human monocyte engraftment and demyelination; variable engraftment between donors.	[114,115]
Humanized PBMC *NSG* mice	NSG mice transplanted with PBMCs from EBV^+^ or relapsing-remitting MS (RRMS) donors. **Disease course and time frame:** Acute/xenogeneic humanized model; disease develops over weeks and is limited by GVHD. **Genetic background:** NSG.	Donor-dependent xenogeneic disease upon EAE induction; PBMCs from EBV-seropositive and RRMS donors drive more severe neurological disease with increased CNS-infiltrating human effector T cells, enhanced T-cell proliferation, and impaired Treg expansion.	Mechanistic insight into EBV-driven immune dysregulation; testing disease-modifying therapies.	Directly models the strong epidemiologic association between EBV and MS; enables evaluation of anti-B-cell and EBV-targeted therapies.	Confounding xenogenic GVHD; limited myeloid reconstitution limits CNS demyelination.	[87]

### 2.3. Type-1 Diabetes

T1D is characterized by immune-mediated destruction of insulin (INS)-producing pancreatic β-cells within the islets of Langerhans, resulting in absolute insulin deficiency and lifelong dependence on exogenous insulin therapy [119,120,121]. Genetic susceptibility is strongly associated with HLA class-II alleles, particularly the haplotypes DRB1*0401-DQB1*0302 and DRB1*0301-DQB1*0201, which shape autoreactive CD4^+^ T-cell responses, while additional non-HLA loci (e.g., those encoding INS, PTPN22, IL2RA, and CTLA-4) contribute to disease susceptibility by modulating thymic selection, Treg function, and peripheral immune activation [119,122,123]. Pathologically, T1D is defined by insulitis due to infiltrating CD4^+^ and CD8^+^ T cells, B cells, macrophages, and dendritic cells (DCs), accompanied by local production of pro-inflammatory cytokines that promote β-cell apoptosis and functional impairment [121,124]. Disease onset is preceded by a preclinical phase marked by the appearance of islet autoantibodies targeting insulin (IAA), glutamic acid decarboxylase (GAD65), insulinoma-associated antigen-2 (IA-2) and zinc transporter 8 (ZnT8), followed by gradual decline in β-cell mass and overt hyperglycemia [119,125]. Despite major advances in understanding T1D genetics and immunopathology, direct analysis of early pancreatic immune events and antigen-specific T-cell responses in humans is still limited. Consequently, a range of murine models from the classical NOD to HLA-transgenic and fully humanized systems have been developed to capture distinct aspects of disease pathogenesis and therapeutic response.

As described in Table 3, T1D models are especially useful for comparing spontaneous autoimmune diabetes, antigen-specific β-cell immunity, and β-cell injury. NOD mice remain the central spontaneous model because they develop insulitis and progressive β-cell destruction in a genetically susceptible background [126,127,128,129,130]. However, disease penetrance is strongly influenced by sex, microbiota, housing conditions, and strain-specific immune regulation, which limits direct translation to human T1D heterogeneity [131]. TCR-transgenic and adoptive-transfer models are powerful for dissecting antigen-specific T-cell activation, tolerance checkpoints, and effector mechanisms, but they may overrepresent selected autoreactive clones [132,133,134,135,136,137]. Streptozotocin-induced diabetes models provide a controlled platform for inducing pancreatic β-cell injury, hyperglycemia, and metabolic dysfunction, and are useful for studying β-cell stress, regeneration, islet transplantation, and diabetes-associated metabolic consequences [138,139]. However, because streptozotocin primarily acts as a β-cell toxin, these models should be interpreted as β-cell injury models rather than models of spontaneous autoimmune initiation. Humanized and HLA-transgenic models are most valuable when the question requires human antigen presentation, patient-derived immune responses, or testing of human-specific immunotherapy systems [140,141,142,143,144,145]. Thus, T1D studies should explicitly distinguish β-cell toxicity, spontaneous autoimmunity, antigen-specific mechanisms, and human immune relevance.

Accurate and standardized evaluation of diabetes and islet pathology is critical for comparing disease progression and therapeutic outcomes in murine models. Diabetes onset is typically defined by sustained hyperglycemia (two consecutive non-fasting blood glucose values ≥ 250–300 mg/dL) together with longitudinal metabolic monitoring. Pancreatic histopathology remains the gold standard, in which formalin-fixed sections are scored in a blinded manner across ≥20–40 islets per mouse using semi-quantitative insulitis scales that distinguish intact islets, peri-insulitis, partial infiltration, and complete destruction [130,147]. Recent standardized workflows incorporating whole-pancreas sectioning, high-resolution imaging, and quantitative morphometry allow assessment of insulitis and β-cell mass [148]. Quantitative measures such as the proportion of infiltrated islets and morphometric assessment of insulin-positive β-cell area provide objective indices of disease severity [130]. Immunostaining for endocrine and immune markers (e.g., insulin, glucagon, CD45, CD4, CD8) defines islet architecture, β-cell loss and immune-cell infiltration [129,149]. Ki67-based proliferation analysis and apoptosis assays further characterize changes in β-cell turnover during disease progression [129,150]. Integration of glycemic measurements, functional islet assays and histological assessment distinguishes impaired β-cell function from progressive immune-mediated β-cell destruction [130,148].

### 2.4. Rheumatoid Arthritis

RA is a chronic, systemic autoimmune disease characterized by persistent synovial inflammation, progressive cartilage destruction, and bone erosion culminating in joint deformity with a global prevalence of approximately 0.5–1% in adults [151,152]. Genetic susceptibility is most strongly linked to HLA-DRB1 alleles encoding the ‘shared epitope’ (a conserved amino acid sequence motif at positions 70–74 of the HLA-DRβ chain), which shape autoreactive CD4^+^ T-cell responses to post-translationally modified autoantigens, especially (but not limited to) citrullinated proteins [153,154,155,156,157]. Additional non-HLA loci include PTPN22, STAT4, CTLA4, TRAF1/C5, and PADI4 that modulate lymphocyte activation, antigen presentation, and cytokine signaling, in addition to environmental exposure (particularly smoking, periodontitis, and gut inflammation) and epigenetic mechanisms [151,158,159]. Clinical RA is preceded by a prolonged preclinical seropositivity for anti-citrullinated protein antibodies (ACPA) and rheumatoid factor (RF), both positively correlated with disease severity [160]. Together, they form immune complexes with citrullinated peptide antigens (e.g., vimentin, α-enolase, fibronectin, fibrinogen, and type-II collagen (CII)), leading to complement activation and progressive synovitis [161]. Although ACPA and RF are strongly associated with RA and may precede clinical disease, their direct pathogenic role in humans remains incompletely established; experimental studies indicate functional heterogeneity, with some ACPA or type-II collagen antibody clones showing arthritogenic activity, others showing no detectable arthritogenicity, and some selected clones even conferring protection in mouse arthritis models [162,163,164]. Histopathologically, the inflamed synovium undergoes marked hyperplasia (infiltration of CD4^+^ T cells, B cells, macrophages, DCs and neutrophils), expansion of fibroblast-like synoviocytes and local production of pro-inflammatory cytokines (i.e., TNF, IL-6, IL-1β, M-CSF and GM-CSF) that promote cartilage damage (via matrix metalloproteinases) and bone erosion (via RANKL-mediated osteoclast differentiation) [161,165,166]. Several mouse models were developed to recapitulate discrete components of RA pathogenesis and to enable preclinical evaluation of targeted immunotherapies, as summarized in Table 4.

RA models vary in the extent to which they reproduce synovitis, autoantibody-mediated inflammation, cartilage destruction, and bone erosion (see Table 4). Antigen-induced models such as collagen-induced arthritis (CIA) [167,168,169,170,171,172], proteoglycan-induced arthritis (PGIA) [177,178,179], and antigen-induced arthritis (AIA) [180,181,182,183,184,185] are useful for studying adaptive immune priming, antigen-specific T- and B-cell responses, cytokine networks, and cartilage-directed joint inflammation. In contrast, collagen antibody-induced arthritis (CAIA) [173,174,175,176] and K/BxN serum-transfer arthritis [186,187,188,189,190] are more appropriate for dissecting autoantibody-, Fcγ receptor-, complement-, neutrophil-, and macrophage-mediated effector mechanisms, because they bypass the initial adaptive priming phase. Spontaneous or genetically driven models, including SKG mice [191,192,193,194], human TNF-transgenic mice [195,196,197,198], and IL-1R antagonist-deficient mice [199,200,201,202], are valuable for studying T-cell tolerance defects, cytokine-driven chronic synovitis, Th17/IL-1/TNF pathways, and genetic susceptibility, although each emphasizes a specific inflammatory mechanism rather than the full heterogeneity of RA. Human HLA- and TCR-based transgenic models (including HLA-DR [203,204,205], HLA-DRB1*0401.AE(o) [206,207], HLA-DQ8 [208,209,210,211,212], HLA + autoantigen [210,213,214,215], TCR-transgenic only [213,216,217], and HLA + TCR-transgenic systems [218] add genetic and antigen-specific relevance by modeling HLA-restricted presentation of arthritis-associated autoantigens and defined autoreactive T-cell responses. Finally, human RA tissue and immune-cell chimera models, including RA synovium/SCID [219,220,221,222,223], RA synovial fibroblast/SCID [219,224,225], RA synovial fluid mononuclear cell/SCID [226,227], Hu-HSC NSG/NOG [228,229], and NSG-RA PBMC-engrafted systems [230], improve translational relevance by incorporating patient-derived synovial tissue, stromal cells, or immune cells, but remain limited by incomplete human tissue architecture, xenogeneic responses, short experimental windows, and donor variability. Therefore, RA models should be interpreted according to whether they primarily represent adaptive immune initiation, autoantibody-mediated effector disease, cytokine-driven inflammation, HLA-restricted antigen specificity, stromal/synovial pathology, or human immune-cell-mediated disease.

Accurate and reproducible assessment of disease pathology in RA animal models requires integration of clinical scoring, histopathology, imaging, and human immune readouts (wherever applicable). In conventional models (e.g., collagen-induced arthritis (CIA), collagen antibody-induced arthritis (CAIA) and other such models), mice are monitored longitudinally and disease severity is quantified using semi-quantitative clinical scoring systems (typically 0–4 per paw) based on erythema, swelling, and joint rigidity, with cumulative scores reflecting total disease burden [168,169,231]. The paw thickness and body weight are commonly recorded as supportive indices of inflammation and systemic disease activity [232]. Histopathologic evaluation remains the gold standard, in which formalin-fixed, decalcified joints are sectioned and scored in a blinded manner for synovial hyperplasia, inflammatory cell infiltration, pannus formation, cartilage degradation (often assessed by Safranin O/Fast Green staining for proteoglycan loss), and bone erosion [168,233]. Standardized Microscopic Arthritis Scoring of Histological sections (‘SMASH’) provides recommendations for a standardized method for processing and characterizing histopathological features of arthritis [234]. Structural imaging using micro-computed tomography (micro-CT) provides quantitative assessment of bone erosion and joint architecture, complementing histological findings [233].

In humanized RA models, including those generated by engraftment of human PBMCs, synovial tissue, or CD34^+^ HSCs, disease scoring should incorporate these conventional endpoints alongside human-specific immunopathological measures, because it is essential to distinguish human immune cell-mediated pathology from residual murine responses in these models [235]. This is often achieved using species-specific markers or depletion strategies, such as detection of human CD45^+^ leukocyte infiltration into joints, characterization of T-cell, B-cell, and myeloid subsets (e.g., CD3, CD4, CD8, CD20, CD68), and quantification of human cytokines (e.g., TNF, IL-6, IL-17) in serum or tissue [230]. In some models, evaluation of autoantibodies such as rheumatoid factor (RF) and anti-citrullinated protein antibodies (ACPA) further supports translational relevance [230,236]. Thus, combined use of clinical indices, detailed joint histopathology, structural imaging, and human immune readouts (if applicable) provides a comprehensive framework for evaluating disease severity, joint destruction, and therapeutic efficacy across both conventional and humanized RA models.

## 3. Influence of the Gut Microbiota on Autoimmune Disease Models

The gut microbiota is an active experimental variable in autoimmune disease models because it can alter immune development, disease penetrance, disease severity, kinetics of onset, and reproducibility across laboratories. Microbiota-dependent variation can arise at several levels, including the presence or absence of defined immunomodulatory taxa, expansion of pathobionts, differences in overall community composition, microbial metabolites, and husbandry-related factors such as vendor source, cage and litter effects, diet, antibiotic exposure, acidified drinking water, co-housing, and germ-free or gnotobiotic housing. Three distinct features of microbial variation independently shape model outcomes. First, individual commensal species can be decisive. Segmented filamentous bacteria (SFB) (e.g., *Candidatus* species) adhere to the ileal epithelium and drive the differentiation of intestinal and systemic Th17 cells, thereby tilting the effector–regulatory balance toward inflammation [237]. Conversely, defined commensals and consortia, including clostridial species and the facultative anaerobe *Enterobacter ludwigii*, expand Treg populations or impose immune tolerance and attenuate disease [232]. Second, microbial metabolites act as soluble effectors of these taxa: short-chain fatty acids (acetate, propionate, and butyrate) generated by bacterial fermentation of dietary fiber promote peripheral Treg induction and reinforce mucosal tolerance [13,14,238]. Third, gross community perturbations (such as reduced diversity, pathobiont blooms, germ-free absence) reset baseline immune tone; and facility/community differences cause divergent model phenotypes [13,14,237].

Microbiota-dependent effects are particularly relevant to the autoimmune models reviewed here. In IBD models, resident enteric bacteria are required for spontaneous colitis in *Il10*^−/−^ mice, and *Helicobacter hepaticus*-induced inflammation alters microbial composition in a strain-specific manner, supporting combined effects of host genotype, immune response, and microbiota composition on colitis susceptibility [57,239]. In MS/EAE models, commensal microbiota can cooperate with myelin autoantigen to trigger spontaneous autoimmune demyelination, and microbiota from patients with MS can modulate T-cell responses and exacerbate EAE-like disease in recipient mice [240,241]. In NOD models of T1D, microbiota and innate immune sensing pathways such as MyD88 influence diabetes development, while sex-dependent microbiome differences can modify autoimmune susceptibility [242,243]. In RA models, SFB can promote autoimmune arthritis through Th17 responses, and microbiota-dependent metabolites such as indole derivatives have been shown to enhance collagen-induced arthritis [244].

Therefore, microbiota should be reported and, where relevant, controlled as part of autoimmune model design. At minimum, studies should report mouse substrain, vendor, facility/barrier status, diet, antibiotic or acidified-water exposure, cage allocation, co-housing or bedding-transfer procedures, and whether mice were maintained under specific-pathogen-free, germ-free, gnotobiotic, or conventional conditions [245]. When microbiota is expected to influence the outcome, investigators should consider littermate controls, cage-level randomization, co-housing before disease induction, defined-flora/gnotobiotic approaches, or microbiota profiling by 16S rRNA sequencing or metagenomics [245]. These practices are especially important when comparing studies across laboratories or interpreting microbiota-sensitive outcomes in IBD, MS, T1D, and RA models.

## 4. Discussion

Murine and humanized mouse models have profoundly shaped current understanding of autoimmune disease pathogenesis by enabling experimental interrogation of tolerance breakdown, autoreactive lymphocyte activation, cytokine networks, tissue injury, and therapeutic responses in vivo. The models reviewed here demonstrate that autoimmune diseases cannot be captured by a single experimental system [1,2,16]. Instead, each model reproduces selected dimensions of disease biology, ranging from acute tissue injury and innate immune activation to chronic spontaneous autoimmunity, antigen-specific effector responses, and human immune-cell-mediated pathology. Therefore, the central challenge in autoimmune disease modeling is not to identify one universally superior model, but to select the model whose biological strengths best match the research question.

**Figure 1:**
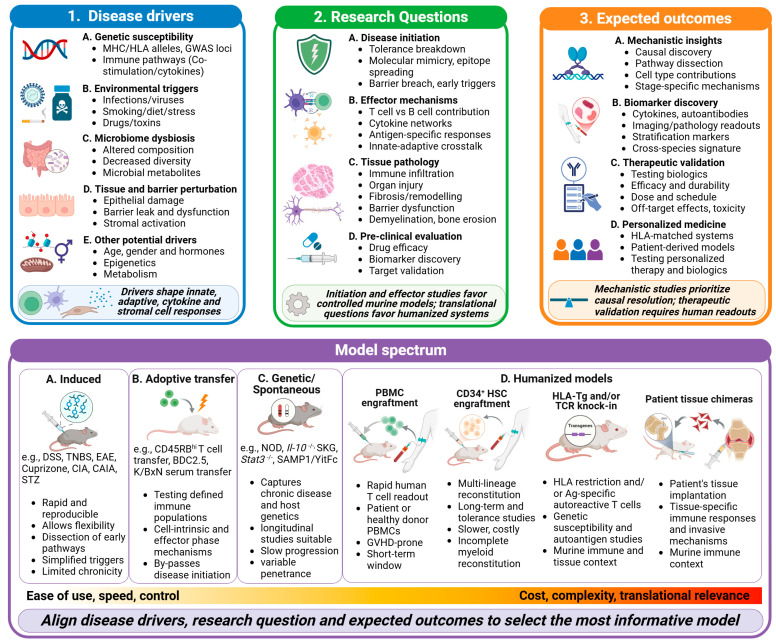
Model selection for autoimmune disease study and therapeutics development.

Across autoimmune diseases, we propose that model selection should be guided by three interconnected considerations: disease drivers, research questions, and expected outcomes (Figure 1). First, the model should reflect the relevant disease driver, including genetic susceptibility, environmental triggers, microbiome dysbiosis, or tissue and barrier perturbation [1,2,16]. For example, chemical and toxin-induced models are useful for controlled injury or inflammatory triggers, but they rarely capture spontaneous breakdown of tolerance. Second, a model should match the research question, whether focused on disease initiation, effector mechanisms, tissue pathology, or preclinical evaluation. For example, adoptive-transfer and TCR-transgenic systems provide causal evidence for defined immune-cell or antigen-specific mechanisms, whereas spontaneous models preserve broader immune-network interactions. Tissue context is also essential because autoimmunity involves epithelial barriers [50,246], endothelial cells and CNS-resident myeloid and glial cells [89,97], β-cells and the islet niche [119,121,124], synovial stromal, endothelial, and myeloid compartments [247], and microbiota [238,240,242,244]. Finally, the selected model should generate the expected outcome, such as mechanistic insight, biomarker discovery, therapeutic validation, or personalized assessment. Humanized, HLA-transgenic, and patient-derived systems can improve translational relevance, but their limitations must be explicitly acknowledged.

Conventional murine models remain indispensable for mechanistic studies because they offer genetic control, reproducibility, experimental tractability, and access to well-defined immunological tools (Figure 1). Induced models such as DSS, TNBS, and oxazolone for IBD, EAE and cuprizone for MS, CIA for RA, and toxin-based models for T1D are particularly useful when the objective is to study defined inflammatory pathways, tissue injury, immune trafficking, or therapeutic response under controlled conditions. Their major advantage is control over experimental timing, dose, and severity. However, many rely on artificial triggers and may not fully reproduce the chronic, multifactorial, and relapsing nature of human autoimmune disease.

Spontaneous and genetically engineered models complement induced systems by allowing disease to arise from host genetic susceptibility, immune dysregulation, and tissue-specific pathology, as exemplified by NOD mice for T1D, *Il-10*^-/-^ and SAMP1/YitFc mice for intestinal inflammation, and SKG and TNF-transgenic models for RA (see respective disease Table 1, Table 2, Table 3 and Table 4). Despite being valuable for longitudinal studies, biomarker discovery, and testing interventions across disease stages, these models remain shaped by strain background, microbiota composition, housing conditions, sex, age, and differences between mouse and human immune pathways. As a result, findings from these models should be interpreted as mechanistic evidence rather than direct clinical validation.

Adoptive-transfer and TCR-transgenic systems provide causal tools for dissecting cell-intrinsic mechanisms. By transferring defined immune populations into recipient mice, investigators can test whether specific T-cell, B-cell, serum antibody, or regulatory cell populations are sufficient or necessary to drive pathology (Figure 1). These models are useful for separating initiation from effector mechanisms, tracking antigen-specific cells, and testing regulatory pathways such as Treg suppression or cytokine dependence. However, they often bypass early disease-initiation events and may overemphasize selected pathways at the expense of broader tissue and environmental complexity.

Humanized mouse models address a major limitation of conventional murine systems by introducing human immune components, HLA-restricted antigen presentation, patient-derived immune cells, or human tissue compartments. PBMC-engrafted models provide rapid access to patient-derived immune activation and donor-specific responses, as exemplified by UC/CD PBMC-humanized colitis models (Table 1) and RA PBMC-humanized arthritis models (Table 4). CD34^+^ HSC-engrafted systems provide broader and more durable human immune reconstitution, whereas BLT-like or thymus-containing systems can improve human T-cell education and HLA-restricted responses. HLA-transgenic, HLA-knock-in, and disease-specific TCR models add value when the research question requires antigen-specific or HLA-restricted mechanisms, while tissue-chimera models are particularly useful for studying human synovial or mucosal microenvironments (Figure 1).

Despite these advantages, humanized models require careful interpretation. PBMC models are limited by xenogeneic GVHD and short experimental windows [248]. CD34^+^ HSC and Bone marrow–Liver–Thymus (BLT) systems are costly, technically demanding, and may show incomplete lymphoid architecture, limited myeloid and innate immune compartments, and imperfect tissue-specific immune education [249,250,251]. Many humanized models contain human immune cells but retain murine stromal, epithelial, endothelial, and cytokine environments [252,253,254]. This mismatch is especially important in diseases wherein tissue barriers, stromal cells, microbiota, or organ-specific niches shape pathology. These limitations are considered in greater detail below under Section 5.

## 5. Limitations of Current Models and Future Directions

**Figure 2:**
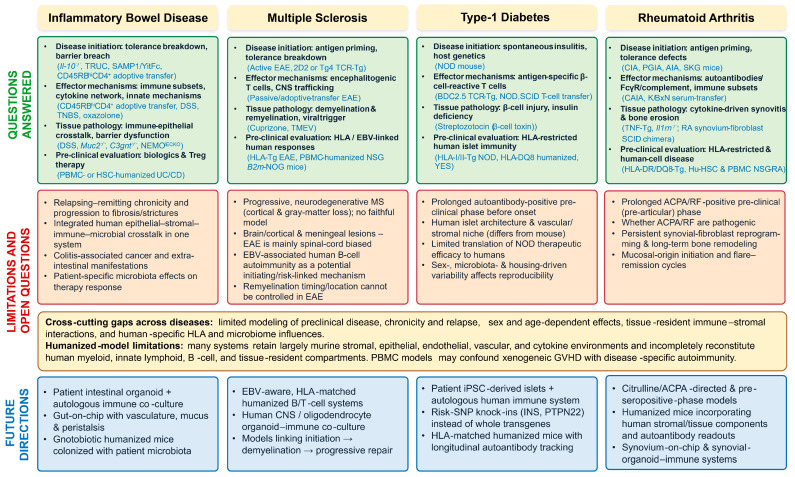
Limitations of current autoimmune disease models and future directions.

Despite their value, the models compiled here collectively leave several immunopathogenic mechanisms incompletely represented, defining clear gaps and opportunities for future model development. Across diseases, current systems poorly capture the prolonged preclinical/asymptomatic phase of human autoimmunity, disease chronicity and relapse, sex- and age-dependent susceptibility, tissue-resident memory and stromal/fibroblast pathology, and the human-relevant contributions of the microbiome, environment, as well as HLA (Figure 2). These gaps are clinically relevant because autoimmune diseases often evolve for years before overt tissue damage becomes apparent. For example, children who develop multiple islet autoantibodies have a high long-term risk of progression to clinical T1D [255], whereas anti-CCP/ACPA can precede the clinical onset of RA by several years [256,257]. Similarly, microbiota composition can reshape immune tone and disease penetrance: segmented filamentous bacteria induce intestinal Th17 cells and can drive autoimmune arthritis in K/BxN mice [244], commensal microbiota cooperate with myelin autoantigen to trigger spontaneous autoimmune demyelination [240], and microbiota-dependent sex-hormone differences regulate diabetes susceptibility in NOD mice [243]. These findings emphasize that autoimmune models must be interpreted not only in terms of the immune pathway they reproduce, but also in relation to the temporal, microbial, hormonal, genetic, and tissue context they omit.

These gaps are also disease-specific. In IBD, DSS, TNBS, and oxazolone models capture epithelial injury, barrier disruption, innate activation, and cytokine-skewed inflammation, but incompletely reproduce relapsing disease, fibrostenosis, patient-specific microbiota, and integrated epithelial–stromal–immune crosstalk. Future models should incorporate controlled microbiota, human intestinal organoids, stromal and immune compartments, and gut-on-chip approaches [245,246,258,259]. In MS, EAE and cuprizone models are useful for inflammatory demyelination and remyelination, respectively, but do not fully capture progressive neurodegeneration, meningeal inflammation, EBV-associated changes in B-cell-mediated pathology, chronic cortical demyelination, or microbiome-dependent CNS autoimmunity. Future MS models would ideally integrate selected combinations of these features according to research question, and several partial attempts are already emerging [86,87,241,260,261,262].

In T1D, NOD and toxin-induced models reproduce insulitis or β-cell injury but incompletely model the prolonged autoantibody-positive phase, HLA-restricted epitope spreading, β-cell stress, and the human islet niche [249]. These limitations are partially addressed with HLA-humanized, antigen-specific models, including HLA-DQ8 or HLA-A2/DQ8 systems incorporating human insulin/preproinsulin or patient-derived insulin-reactive TCRs to study human-relevant islet antigen recognition and HLA-restricted T-cell responses [143,144,146]. Complementary human stem-cell and islet-based platforms, including patient-derived endocrine cells combined with autologous immune cells, are beginning to model β-cell stress and immune–β-cell interactions in a more human tissue context [263]. However, these approaches will likely need to be combined with longitudinal autoantibody monitoring, human islet vascular/stromal niches, and patient-specific immune and genetic backgrounds to better reproduce the stepwise progression of human T1D. In RA, collagen-induced arthritis, K/BxN serum transfer, SKG, and cytokine-transgenic models capture selected inflammatory, antibody-mediated, T-cell-driven, or cytokine-driven pathways but incompletely represent preclinical seropositivity, mucosal initiation, flare–remission cycles, persistent synovial fibroblast reprogramming, and long-term bone remodeling. Some of these gaps are being addressed by single-cell-informed studies, 3D spheroids and synovium-on-chip approaches that better capture mucosal autoimmunity and immune–vascular–fibroblast crosstalk in RA [247,264,265].

Humanized models, although increasingly powerful, still rely largely on murine stromal, epithelial, endothelial, vascular, and cytokine environments and incompletely reconstitute human myeloid, innate-lymphoid, B-cell, and tissue-resident compartments [252,253,254]. A central caveat for humanized models, applicable across all four diseases, is that the presence of human immune cells does not by itself constitute disease-specific human autoimmunity. Human PBMC-engrafted mice are prone to xenogeneic graft-versus-host reactivity, so much of the observed inflammation may reflect human anti-mouse xenoreactivity rather than antigen-specific autoimmune pathology [248,266]. Therefore, positive findings should be interpreted cautiously and, ideally, anchored to disease-specific, antigen-restricted readouts. Equally important, incomplete human myeloid, stromal, vascular, epithelial, and glial compartments limit conclusions about tissue-specific mechanisms: a humanized model may faithfully report a human T- or B-cell response yet remain uninformative about the epithelial, synovial, islet, or CNS niche in which human disease unfolds [247,253,254]. In practical terms, humanized systems are well suited to questions about human lymphocyte specificity, HLA-restricted antigen recognition, donor stratification, and the in vivo activity of human-targeted biologics, but remain limited for questions that depend on a fully human tissue microenvironment.

Both cell-transfer and genetically humanized models introduce artifacts that warrant explicit consideration. In PBMC-humanized mice, mature human T cells that were selected on human HLA in the donor thymus can recognize recipient murine MHC and tissues, causing human anti-mouse xenoreactivity and GVHD [248,266]. Whereas, in HSC-humanized mice lacking human thymic tissue or appropriate HLA expression, newly generated human T cells develop within a murine thymic environment, resulting in imperfect HLA restriction and selection [249,250,252]. Genetically humanized models may also exhibit ectopic or supraphysiological transgene expression, the presence of competing murine orthologs, and disrupted human–mouse molecular interactions [252,253,254]. These artifacts can be reduced through targeted knock-in or gene replacement under endogenous regulatory control and coordinated humanization of interacting components, such as HLA together with compatible human coreceptors and accessory molecules [203,204,252,253].

Future models are likely to address these gaps through several converging strategies. These include but are not limited to (a) next-generation humanized mice with human cytokine and growth-factor knock-ins (e.g., MISTRG and NSG-SGM3 background models) to support human myeloid and innate immune development [254,267,268]; (b) thymus-containing systems, including BLT and neonatal thymus [267,269], and patient-specific iPSC-derived thymic organoids [270] to improve HLA-matched T-cell education; (c) gnotobiotic humanized systems colonized with patient-derived microbiota [271]; and (d) complementary, reductionist alternatives like in vitro/ex vivo human organ-on-chip [246,258,272] or organoid-immune co-culture platforms (for co-engraftment of human target tissue (intestinal/synovial/islet/CNS) organoids with an autologous human immune system [273]) to recreate disease-relevant niches. We summarize current limitations and these future directions schematically in Figure 2.

## 6. Conclusions

A critical interpretation of animal models should therefore avoid asking whether a model is ‘good’ or ‘bad’ in general. A more useful question is: what component of disease does this model reproduce, what component does it omit, and what conclusion can be reasonably drawn from it? A model may be excellent for testing cytokine blockades but weak for studying disease initiation, whereas another may reproduce tissue pathology but lack human antigen presentation. This question-driven framework allows murine and humanized models to be used as complementary tools rather than competing approximations of human disease. Concrete examples illustrate this framework. The K/BxN serum-transfer and CAIA models are well suited to testing therapies targeting antibody-, FcγR-, and complement-driven effector inflammation but provide limited information about loss of tolerance, for which spontaneous/genetic models, such as NOD or SKG mice, and antigen-specific TCR-transgenic systems are more appropriate. DSS colitis is ideal for epithelial repair and innate immune questions yet inappropriate for studying adaptive T-cell tolerance, which is better addressed by the CD45RB^hi^CD4^+^ transfer model. Cuprizone is well suited to studying demyelination and remyelination, whereas EAE is more appropriate for evaluating immunomodulatory disease-modifying therapies. PBMC-humanized models can demonstrate engagement of human T cells in vivo but cannot, by themselves, establish disease-specific autoimmunity because substantial inflammation may reflect xenogeneic reactivity. Thus, the relevant question is never whether a model is good or bad, but which disease component it reproduces and what conclusion it can legitimately support.

Finally, the usefulness of any animal model for studying human autoimmune disease depends not only on its biological design but also on the rigor with which experiments are conducted and reported. Surveys of preclinical literature show that adherence to recommended standards remains low. In a recent analysis of 120 comparative animal studies, none employed a fully valid, unbiased design under the authors’ criteria (estimated prevalence 0%; 95% CI, 0–2.5%) [274]. Common deficiencies included failure to control cage effects, incomplete randomization and blinding, and incorrect identification of the experimental unit or unit of analysis. Model selection should therefore be accompanied by appropriate power and sample-size calculations, randomization, blinded outcome assessment, control of cage and environmental effects, and preregistration where feasible. Experiments should also be reported completely in accordance with the ARRIVE 2.0 guidelines [275]. Such rigor is a major determinant of the reproducibility and translational reliability of findings from animal models.

## Figures and Tables

**Table 1:** Animal models for Inflammatory Bowel Disease.

Model	Description	Phenotype and Pathology	Applications	Advantages	Limitations	References
***Murine Models***						
Dextran sodium sulfate (DSS) colitis model	DSS (a sulfated polysaccharide) given in drinking water disrupts tight junctions causing damage to colonic epithelium and permeability to luminal antigens and microbes. **Disease course and time frame:** Acute colitis (single DSS cycle) 5–10 days; chronic disease (3–4 cycles) over ~4–8 weeks. **Genetic background:** C57BL/6 (highly susceptible) and BALB/c.	UC-like acute mucosal inflammation with diarrhea, erosions/ulcers, loss of crypts and goblet cells; innate cell-mediated with progressive recruitment of CD4^+^ and CD8^+^ T cells; T and B cells not required for induction but shape chronicity over time; epithelial barrier is gradually repaired after acute exposure while chronic exposure leads to epithelial remodeling and tumorigenesis; chemokines (MIP-3α Eotaxin, KC/CXCL1, MCP-1, MIG, IP-10 and MIP-3α) and cytokines (IL-1α, IFN-γ, IL-6, TNF-α. IL-1β and IFN-γ) expressed in acute phase, while chronic phase also has elevated RANTES, BLC and IL-17A.	Study epithelial barrier injury, innate responses (e.g., TLR signaling), host–microbiome interactions and role of cytokines; mechanisms of acute vs. chronic colitis, fibrosis and colitis-induced colorectal cancer; testing therapeutic treatments for IBD.	Simple, rapid, inexpensive and reproducible; disease severity can be controlled by DSS dose and duration.	Weak adaptive immune components; does not model complex pathogenesis seen in human IBD (tolerance breakdown or antigen-specificity); model severity can vary substantially with mouse strain and microbiota composition.	[31,32,33,34,35,36,37]
Trinitrobenzene Sulfonic Acid (TNBS) colitis model	TNBS (administered intrarectally in ethanol) acts as a hapten to bind tissue or microbiota-associated proteins, rendering them immunogenic and provoking a T-cell-mediated response. **Disease course and time frame:** Acute ~3–7 days; chronic with repeated low-dose administration over weeks. **Genetic background:** SJL/J and BALB/c are susceptible, while C57BL/6 is relatively resistant.	Crohn’s-like transmural colitis with ulceration, goblet-cell loss, diarrhea, weight loss, and rectal prolapse; Th1-driven responses (with infiltration of CD4^+^ T cells, neutrophils, and macrophages in lamina propria); pathology mediated by TNFα, IL-12, IFNγ and IL-17 (although IFN-γ is considered a weaker mediator); recent data show increased α-synuclein aggregates in colonic mucosa and enteric nervous system.	Study intestinal inflammation, mucosal immune responses, barrier injury, cytokine pathways, fibrosis, and preclinical therapeutic efficacy; host–microbiome interactions.	Simple, rapid, inexpensive; reproducible if protocol variables are controlled; produces robust inflammation with features that resemble transmural Crohn’s-like colitis; disease severity can be controlled by TNBS dose and duration.	Does not model complex pathogenesis seen in human IBD; outcomes vary with strain, species, TNBS dose, ethanol concentration, and administration schedule; distal-colon predominant disease due to rectal delivery.	[38,39,40,41]
Oxazolone Colitis model	Oxazolone (intra-rectal delivery) haptenizes host proteins and disrupts epithelial barrier, triggering a T-cell-mediated hypersensitivity response leading to epithelial injury and barrier dysfunction. **Disease course and time frame:** Acute onset 1–3 days, typically resolves within ~1–2 weeks; chronic with repeated dosing. **Genetic background:** SJL/J and BALB/c susceptible; C57BL/6 relatively resistant (pre- sensitization required).	UC-like colitis characterized by superficial mucosal inflammation, ulceration, goblet cell depletion, edema, and colon shortening; presence of mixed inflammatory infiltrate (lymphocytes, neutrophils, eosinophils). Th2-driven, NKT cell and IL-13-mediated epithelial damage and increased permeability; persistent inflammation with repeated exposure.	Modeling UC-like disease; studying Th2/NKT-cell-mediated immunity; investigating IL-13-driven epithelial barrier dysfunction; evaluating therapies targeting type-2 cytokines; mechanisms determining epithelial integrity and mucosal immunity.	Closely mimics immunopathogenesis of UC (Th2/IL-13 axis); reflects role of NKT cells, epithelial barrier dysfunction and cytokine-driven mucosal injury; mechanistically distinct from DSS and TNBS colitis.	Strong strain dependence (e.g., C57BL/6 are relatively resistant and may require pre-sensitization); does not model complex pathogenesis seen in human IBD; disease often acute and distal colon-restricted.	[42,43]
*Muc2*^-/-^ mice	Genetic loss of Mucin-2 (Muc2, a primary gastrointestinal mucin) leads to defective mucus barrier. **Disease course and time frame:** Spontaneous colitis from ~5 weeks of age; neoplasia later in life. **Genetic background:** 129Sv shows early/overt colitis around weaning compared to C57BL/6.	Spontaneous, progressive disease (with features resembling active UC) including goblet cell loss, crypt hyperplasia with colorectal hyperplasia and adenomas developing at end stages; microbiota-dependent inflammation associated with increase in colonic neutrophils, T cells and macrophages; significant increase in IL-6, TNF-α, IL-1β and IKKβ; DSS treatment aggravates disease severity.	Barrier dysfunction; microbial-epithelial crosstalk and innate-adaptive immune system crosstalk.	Spontaneous disease; strong epithelial relevance.	Genetic etiology; neoplasia later in life complicates long studies.	[44,45,46,47]
*C3gnt*^−/−^ mice	Mice lacking C3GnT (β1,3-N-acetylglucosaminyltransferase), an enzyme involved in the synthesis of all core 3-derived O-glycans in intestinal mucins; defective mucin glycosylation impairs mucus barrier integrity. **Disease course and time frame:** Chronic barrier defect; overt colitis only upon additional trigger (e.g., DSS). **Genetic background:** C57BL/6 or C57BL/6J × 129/SvImJ.	Increased intestinal permeability and reduced Muc2 levels, resulting in a compromised mucus barrier; do not typically develop severe spontaneous colitis but show marked susceptibility to chemically induced colitis (e.g., DSS) and colorectal tumorigenesis; enhanced immune activation upon microbial contact with the epithelium.	Study mucus barrier function and mucin glycosylation in intestinal homeostasis; host–microbiota interactions; modeling susceptibility to colitis and colitis-associated cancer.	Directly models epithelial barrier defects and mucin-associated pathology; can be combined with other models (e.g., DSS) for mechanistic studies.	No spontaneous colitis as it requires additional triggers (e.g., DSS) to induce inflammation; does not model complex pathogenesis seen in human IBD.	[48,49,50]
TRUC mice	*T-bet*^-/-^ x *Rag2*^-/-^ mice; T-bet restricts TNF-α synthesis by DCs to prevent immune responses against commensal microbes; T-bet deletion (in *Rag2*^-/-^ mice) produces spontaneous, aggressive colitis resembling human UC. **Disease course and time frame:** Spontaneous, highly penetrant colitis by ~4 weeks of age; strongly microbiota-, co-housing-, maternal-transmission-, and facility-dependent. **Genetic background:** BALB/c.	Spontaneous, highly penetrant colitis by 4 weeks of age with increasing severity; continuous inflammation of the rectum; inflammatory infiltrate in lamina propria and goblet cells, crypt loss and increased epithelial permeability; microbiota and TNF-α (by DCs) drive disease; colitogenic microbiota from TRUC mice can transmit disease to *T-bet*-sufficient mice; chronic TRUC colitis is IL-7Rα^+^ innate lymphoid cells (ILCs)-dependent.	Study innate immunity-driven colitis; host–microbiota interactions; modeling microbiota transmissibility of disease; testing therapies targeting innate immune pathways and microbiota.	Spontaneous colitis; strongly demonstrates role of innate immunity independent of adaptive immune cells; unique model for microbiota-driven and transmissible disease; highly relevant for studying TNF-mediated inflammation.	Represents a specific mechanistic pathway (T-bet deficiency) rather than broad IBD heterogeneity; strong dependence on microbiota composition (variability across facilities); does not model complex pathogenesis seen in human IBD.	[61,62]
NEMO^IEC-KO^ mice	Intestinal epithelial-specific deletion of NEMO (also known as IKK-γ, an essential regulatory subunit of NF-κB); NEMO prevents intestinal inflammation by inhibiting RIPK1 kinase activity-mediated IEC death. **Disease course and time frame:** Chronic, spontaneous colitis from early age (within weeks). **Genetic background:** *Ikbkg/Nemo^fl^*^/fl^ × *Villin*^Cre^, on a C57BL/6 background.	Epithelial cell apoptosis and barrier breakdown; loss of antimicrobial peptide production (Paneth cell dysfunction); bacterial translocation and microbiota-driven inflammation; severe colonic inflammation with immune-cell infiltration; disease is TNF-dependent and driven by dysregulated host–microbiota interactions; IFN-γ and IL-17 responses seen upon specific pathogen exposure.	Study epithelial barrier dysfunction and microbiota-driven inflammation; investigate NF-κB signaling in intestinal homeostasis; model TNF-mediated colitis mechanisms and preclinical testing of anti-TNF therapies.	Develops spontaneous colitis (no chemical induction); strongly demonstrates role of epithelial NF-κB signaling in disease prevention; captures host–microbiota interactions central to IBD; mechanistically relevant to human NEMO mutations associated with colitis.	Pathology driven primarily by epithelial defects but not full immune dysregulation; strong dependence on TNF signaling and microbiota composition; does not fully capture adaptive immune complexity of human IBD; severe phenotype may limit chronic modeling.	[51,52,53,54]
*Il-10*^-/-^ mice	Genetic loss of IL-10 (an anti-inflammatory cytokine) promotes immune reactivity to microbial antigens and triggers colitis. **Disease course and time frame:** Chronic, spontaneous colitis in ~8–12 weeks. **Genetic background:** C3H/HeJBir, 129/SvEv, and BALB/c backgrounds show highest severity; 129 × C57BL/6J hybrids show intermediate while C57BL/6J show mild disease.	Enteric microbiota-induced spontaneous chronic colitis; activated Th1 (IL-12/IFN-γ) and Th17 (IL-23/IL-17) pathways with increased expression of IL-22 by Th17 and ILC3 cells in the small bowel; strain-specific colitis susceptibility is dependent on gut microbial composition.	Effect of microbial dysbiosis; Cytokine-driven inflammation; immune tolerance mechanisms.	A ‘multi-hit’ model to study role of genetics, immune cells, and microbiota in colitis pathology; evaluate therapeutic agents and antibiotics.	Genetic background and microbiota determine disease penetrance severity; lack of Il-10 limits studying role of Tregs.	[55,56,57,58]
*Stat3*^-/-^ mice	Myeloid-specific *Stat3*^-/-^ disrupts IL-10-mediated anti-inflammatory signaling in myeloid cells, causing unregulated inflammatory cytokine production upon LPS induction; Stat3/IL12p40 and Stat3/TLR dual KO follow-up models provide mechanistic insights. **Disease course and time frame:** Chronic, spontaneous; enterocolitis within the first weeks to months of life. **Genetic background:** Myeloid-specific *LysM*^Cre^ *Stat3*^fl/fl^ mice; commonly described on a C57BL/6-derived or mixed C57BL/6 × 129/Sv background.	Spontaneous chronic enterocolitis with exaggerated inflammatory cytokines (including TNF-α, IL-1, IL-6 and IFN-γ), increased susceptibility to endotoxin shock, and Th1-skeweed immune responses; Deletion of IL-12p40 (IL-12p40/Stat3 dual KO) normalizes Th1 responses and prevents colitis indicating IL-12p40 is essential while TLR4/Stat3 dual KO show reduced IFN-γ production by T cells supporting a role for microbial/TLR4-driven myeloid activation.	Study role of IL-10–STAT3 signaling in myeloid cells, macrophage/neutrophil deactivation, crosstalk between microbiota-induced Th1-driven intestinal inflammation; testing IL-12/IL-23, TLR4, and T-cell-dependent mechanisms in IBD-like inflammation.	Spontaneous disease; mechanistically strong model linking defective myeloid anti-inflammatory signaling to chronic colitis; helps distinguish essential versus non-essential inflammatory mediators.	Cell-type restricted mechanism; primarily models loss of IL-10 responsiveness in innate immune cells rather than the full multifactorial pathogenesis of human IBD; disease severity may depend on microbial exposure and inflammatory tone.	[59,60]
*SAMP1/YitFc* mice	Spontaneous ileitis-prone subline of senescence-accelerated mouse P1/Yit strain; develop chronic intestinal inflammation under SPF conditions without chemical, genetic, or adoptive-transfer manipulation. **Disease course and time frame:** Chronic, spontaneous CD-like ileitis by ~10 weeks of age, progressive thereafter. **Genetic background:** Inbred SAMP1/YitFc sub-line derived from the senescence-accelerated mouse P1/Yit strain.	Spontaneous, discontinuous CD-like ileitis involving the terminal ileum and caecum at ~10 weeks; intestinal wall thickening, villous atrophy, crypt hyperplasia, goblet cell depletion and inflammatory infiltrates; increased neutrophils, macrophages, CD3ε^+^ T cells, myeloperoxidase activity, and inducible nitric oxide synthase expression.	Study spontaneous CD-like ileitis, host–microbiota interactions, early events, epithelial barrier dysfunction, leukocyte trafficking, chemokine networks; Th1/TNF-mediated inflammation; fibrosis, and therapeutic interventions for chronic ileitis.	Spontaneous ileal inflammation without exogenous induction; closely resembles human CD in location, chronicity, and histopathology; understanding mechanisms of disease initiation and chronicity.	Primarily reflects but not full model CD; disease severity depends on microbiota, housing conditions, subline, and genetic background; not driven by a defined single genetic lesion; slower onset than induced models.	[63,64]
CD45RB^hi^CD4^+^ T-cell transfer model	Naive CD45RB^hi^CD4^+^ T cells from immunocompetent donors transferred into lymphopenic recipients (*Rag1/2*^-/-^ or SCID) expand in response to commensal microbial antigens and differentiate into pathogenic effector T cells. **Disease course and time frame:** Chronic. Colitis develops ~5–8 weeks after T-cell transfer. **Genetic background:** Requires syngeneic donor and recipient pairing (commonly C57BL/6 CD4^+^CD45RB^hi^ donor cells into C57BL/6 *Rag1/2*^−/−^ recipients, or BALB/c donor cells into CB17-SCID/BALB/c recipients.	Chronic T-cell-mediated colitis with progressive weight loss, diarrhea, colon thickening, epithelial hyperplasia, goblet-cell loss, crypt distortion, and dense infiltration by activated CD4^+^ T cells and macrophages; microbiota-dependent disease driven mainly by Th1/Th17-type responses, with high IFN-γ, TNF-α, IL-17; co-transfer of CD4^+^CD25^+^FOXP3^+^ Tregs suppresses colitis.	Study T-cell-intrinsic mechanisms of intestinal inflammation, peripheral tolerance, Treg-mediated suppression, effector T-cell differentiation, cytokine signaling, host–microbiota interactions; testing genes or pathways in donor T cells; evaluating Treg-based or cytokine-targeted therapies.	Highly reproducible and mechanistically clean model of adaptive immune-mediated colitis; allows direct manipulation of transferred T-cell populations; widely used to define Treg function.	Strictly dependent on microbiota composition and housing conditions; does not capture genetic susceptibility or epithelial barrier defects; may overemphasize T-cell-driven mechanisms while underrepresenting epithelial, stromal, innate immune, and environmental contributions.	[65,66,67,68]
***Humanized mouse models***						
UC PBMChumanized NSG mice	NSG (common name NOD scid gamma) mice reconstituted with PBMCs derived from UC patients followed by a trigger (e.g., ethanol) to induce colitis. **Disease course and time frame:** Acute, xenogeneic window ~2–4 weeks; limited by GVHD. **Genetic background:** NSG (NOD.Cg-*Prkdc(scid) Il2rg(tm1Wjl)*/SzJ).	Patient-derived immune cells (CD4^+^/CD8^+^ T cells, monocytes/macrophages) infiltrate the colon and drive inflammation (with cytokine signatures such as IFN-γ), edema, crypt elongation, tufting, fibrosis and strictures.	Assessing patient-specific immune responses; study cytokine networks and Treg functions; biomarker discovery; evaluation of biologics (e.g., anti-TNF therapies).	Captures donor-specific inflammatory signatures; useful for testing therapeutics that target human immune molecules; can support personalized or stratified preclinical testing.	Risk of xenogeneic graft-versus-host-disease (GVHD); incomplete myeloid compartment; absence of human intestinal epithelium; requires external trigger (e.g., ethanol).	[69,70,71,72]
CD PBMC- humanized NSG mice	NSG mice reconstituted with PBMCs from CD patients and then challenged to induce intestinal inflammation. **Disease course and time frame:** Acute, ~2–4 weeks; limited by GVHD. **Genetic background:** NSG (NOD.Cg-*Prkdc(scid) Il2rg(tm1Wjl)*/SzJ).	CD-associated inflammatory features with donor-dependent immune activation, influx of human leukocytes, inflammatory edema, altered cytokine profiles, and intestinal tissue remodeling; model partially reflects CD-like inflammation but does not fully reproduce transmural granulomatous CD.	Compare UC- and CD-derived immune signatures; examine mechanisms underlying patient-specific inflammatory processes; evaluate cytokine pathways and therapeutic responses in CD.	Uses patient-derived CD immune cells; enables comparison between UC and CD models within the same NSG platform.	Does not fully recapitulate chronic transmural Crohn’s disease, granuloma formation, stricturing, human intestinal stroma, or microbiome complexity; GVHD and donor variability; still dependent on external challenge.	[72,73]
HSC humanized TNBS-induced colitis with low-dose IL-2	NSG mice reconstituted with healthy human CD34^+^ HSCs sensitized with TNBS and treated with daily low-dose IL-2 or PBS, followed by rectal TNBS challenge. **Disease course and time frame:** Acute colitis within weeks of TNBS sensitization after engraftment. **Genetic background:** NOG (NOD.Cg-*Prkdc(scid) Il2rg(tm1Wjl)*/SzJ).	Acute colitis with weight loss, colon shortening, and histologic inflammation; low-dose IL-2 accelerated recovery from weight loss, prevented higher disease activity index and colon shortening; expanded human Tregs in blood and spleen, and reduced human IL-12 levels in colon.	Study peripheral human Treg expansion and IL-2 responsiveness in experimental colitis; evaluate low-dose IL-2 as an immune-regulatory therapy for IBD; rapid preclinical testing of therapies targeting human Treg expansion or function.	Demonstrates role for low-dose IL-2 therapy in Treg expansion and protection from colitis; shows measurable clinical, histologic, cellular, and cytokine endpoints.	Does not fully model UC or CD; inflammatory trigger is not entirely human immune-cell dependent; protection by low-dose IL-2 requires human immune cells but may also involve other mouse innate cells.	[74]
PBMC- humanized DSS-induced Colitis	*NOG* (common name NOD/Shi-scid IL2Rγnull) mice reconstituted with healthy human donor PBMCs subsequently exposed to DSS to induce colitis; simultaneous injection of autologous Tregs isolated and expanded from the same donor PBMCs. **Disease course and time frame:** Acute DSS colitis, ~1–2 weeks after DSS treatment in engrafted mice. **Genetic background:** NOG (NOD.Cg-*Prkdc(scid) Il2rg(tm1Sug)*/JicTac).	Acute DSS colitis with weight loss, diarrhea, intestinal bleeding, colon shortening, epithelial injury, crypt damage, and human inflammatory cell infiltration; Autologous Treg treatment reduces disease activity index, preserves colon length, decreases histological inflammation and decreases hCD3^+^ infiltration.	Study human immune-cell contribution to DSS-induced intestinal inflammation; evaluate personalized/autologous Treg-cell therapy for IBD; test human-specific cellular immunotherapies and immune-modulatory drugs.	Combines DSS-induced epithelial injury with human PBMC engraftment; enables human-specific immune readouts; provides a translational platform for evaluating personalized Treg therapies.	Does not fully model UC or CD; limited reconstitution of human myeloid, B-cell, epithelial, stromal or microbiome compartments; risk of xenogeneic GVHD; does not model patient-specific disease.	[75]
CD4^+^ T-cell-humanized NSG-Aβ°DR1 mice	NSG-Aβ°DR1 mice (lack murine MHC class II and express human HLA-DR1) reconstituted with human CD4^+^ T cells followed by rectal TNBS challenge; The model was used to test Treg induction via activation of aryl hydrocarbon receptor (AHR) to suppress colitis. **Disease course and time frame:** Acute, human CD4^+^ T-cell-driven TNBS colitis over ~1–2 weeks. **Genetic background:** NSG (NOD.Cg-*Prkdc(scid) Il2rg(tm1Wjl)*/SzJ).	TNBS-induced colitis driven by human CD4^+^ T cells with body-weight loss, histologic colonic inflammation with human CD3^+^ T-cell infiltration and cytokines (i.e., TNF and IFN-γ). AHR agonist treatment ameliorated colitis via increased suppressive capacity of human Tregs (via CD39, granzyme B, FOXP3, and IL-10).	Study human CD4^+^ T-cell-mediated intestinal inflammation; evaluate human Treg induction and suppressive mechanisms in vivo; test AHR activation as an immune-tolerance-promoting therapeutic approach for IBD.	Mechanistically clean human T-cell-driven colitis; enables assessment of CD4^+^ T-cell responses in context of HLA-DR1; directly tests human immune-regulatory pathways in vivo; test pharmacologics to improve Treg function.	No spontaneous UC/CD; driven mainly by transferred human CD4^+^ T cells–does not fully model the broader immune, epithelial, stromal, microbial, and vascular complexity of IBD; utilizes a selected HLA-DR1 context.	[76]

**Table 3:** Animal models for Type-1 diabetes.

Model	Description	Phenotype and Pathology	Applications	Advantages	Limitations	References
***Murine Models***						
NOD mice	Spontaneous T1D (~12 weeks of age, higher incidence in females); insulin essential autoantigen. **Disease course and time frame:** Chronic, spontaneous; insulitis from ~3–4 weeks; overt diabetes ~12–30 weeks, earlier/higher in females. **Genetic background:** NOD/ShiLtJ or related NOD sub-strains; higher susceptibility with MHC-II (H-2g7 haplotype) and 20 non-MHC insulin-dependent diabetes (Idd) loci.	Extensive islet immune infiltrates consisting of autoreactive CD4^+^ and CD8^+^ T cells and B cells (producing anti-insulin antibodies), besides DCs and macrophages; defective Tregs and macrophage maturation; low levels of natural killer (NK) cell activity, C5a and hemolytic complement.	Studying disease pathogenesis and influence of candidate genes (using KO and Tg mice) and environmental factors including diet, microbiome, and infections; investigating both effector and regulatory immune mechanisms.	Gold standard—shares several immunogenetic and pathological features with human T1D; highly amenable to genetic modification and adoptive transfer studies.	Rapid disease onset; lack of complete translatability to human T1D for disease intervention; gender bias.	[126,127,128,129,130]
BDC2.5 TCR Tg NOD mice	NOD mice expressing diabetogenic CD4^+^ T-cell receptor reactive to a hybrid insulin-chromogranin A peptide (presented by the NOD MHC-II molecule I-Ag7). **Disease course and time frame:** Peri-insulitis ~3 weeks; diabetes onset variable/accelerated. **Genetic background:** NOD/ShiLtJ.	Early peri-insulitis starting ~3 weeks; high frequency of islet-reactive CD4^+^ T cells; aggressive diabetes after adoptive transfer or when crossed to NOD.SCID or NOD.*Rag2*^-/-^ mice.	Antigen specificity and immune tolerance studies; co-stimulation and checkpoint pathways; disease progression kinetics; therapeutic testing and immune intervention.	Defined antigen specificity; high reproducibility due to rapid synchronized diabetes; reductionist model for genetic dissection of disease progression.	Artificially high autoreactive cell frequency; pathology driven by CD4^+^ T cells while human T1D involves CD8^+^ T cells too; does not fully model chronic, stochastic β-cell loss.	[132,133,134]
NOD.SCID or NOD.*Rag2*^-/-^ mice	NOD mice lacking functional T/B cells; induced T1D upon transfer of diabetic NOD splenocytes or pathogenic T cells (e.g., BDC2.5 or NY8.3). **Disease course and time frame:** Diabetes onset ~2–6 weeks after transfer of diabetogenic cells. **Genetic background:** NOD.SCID or NOD.*Rag2*^-/-^ recipients.	Absence of spontaneous insulitis due to lack of T/B cells from SCID or *Rag2* deficiency; transfer of diabetogenic T cells or splenocytes induces synchronized autoimmune insulitis, beta-cell destruction, hyperglycemia, and diabetes.	Adoptive-transfer studies; testing diabetogenic potential of T cells; antigen-specific therapy; Treg functions.	Highly reproducible and synchronized disease; retains NOD *Idd* susceptibility.	Lack of spontaneous disease limits studying initial kinetics; limited relevance for environmental triggers.	[135,136,137]
Streptozotocin-induced diabetes model	Chemically induced β-cell injury model generated by single high-dose or multiple low-dose streptozotocin (STZ), a glucose analog preferentially taken up by pancreatic β-cells through GLUT2 glucose transporter. **Disease course/time frame:** acute toxin-induced diabetes; hyperglycemia typically develops within days to ~1–2 weeks depending on dose, strain, sex, age, and protocol. **Genetic background:** DBA/2 is most sensitive, followed by C57BL6 and CD-1, while BALB/c mice are resistant.	Dose-dependent pancreatic β-cell damage, insulin deficiency, hyperglycemia, weight loss, glycosuria, and metabolic dysfunction. Low-dose protocols show secondary islet inflammation, but the initiating event is direct β-cell toxicity and not spontaneous autoimmune priming.	Used to study β-cell injury, β-cell stress, insulin deficiency, hyperglycemia, regeneration, glucose homeostasis, diabetes-associated metabolic consequences, and experimental β-cell/islet replacement approaches.	Rapid, inexpensive, reproducible, and experimentally controllable; diabetes onset and severity can be adjusted by dose and injection schedule.	Artificial chemical-injury model; does not model spontaneous autoimmune initiation, preclinical autoimmunity, antigen-specific tolerance breakdown, or the full chronic immune pathogenesis of human T1D; strain-, sex-, age- and dose-dependent variability requires careful interpretation.	[138,139]
***Humanized mouse models***						
NOD-cMHC-I/II^-/-^ mice	NOD mice lacking mouse MHC-I/II expression; allow transgenic expression of any HLA-I/II allele combinations (e.g., HLA-A2 and HLA-DQ8) as well as patient-derived TCRs (e.g., 20D11, 6H9). **Disease course and time frame:** Induced/TCR-transfer dependent; no spontaneous diabetes without compatible HLA/TCR system. **Genetic background:** NOD/ShiLtJ with cMHC-I/II deficiency.	No spontaneous insulitis; HLA-restricted TCR-driven insulitis; TCR intrinsic properties determine pathogenicity; transfer of insulitis into compatible recipients.	Determine pathogenic potential of patient TCRs; mechanistic dissection of HLA-restricted autoimmunity; development of antigen-specific therapy.	Modular system allows combination of different HLAs, TCRs and antigens; lack of mouse MHC-I/II improves signal-to-noise ratio.	No spontaneous T1D; presence of murine immune system; restricted HLA-I/II diversity does not model human heterozygosity.	[140,146]
HLA-I Tg NOD.β2m^-/-^ mice and HLA-I Tg NOD.cMHC-I^-/-^ mice	NOD.β2m^-/-^ (NOD with β2m^null^ mutation) or NOD.cMHCI^-/-^ (NOD with CRISPR removal of *H2-K^d^* and *H2-D^b^*) mice with expression of human HLA class-I transgenes (e.g., HLAA2, A3 and B7). **Disease course and time frame:** Kinetics broadly like NOD depending on HLA-I transgene. **Genetic background:** NOD/ShiLtJ with β2m or cMHC-I deficiency.	Spontaneous diabetes mediated by HLA-I-restricted CD8^+^ T cells; Classical NOD-like disease (progressive insulitis, β-cell destruction, hyperglycemia).	Identifying HLA-I-restricted epitopes of insulin; testing of patient HLA risk alleles; role of CD8^+^ T cells in T1D pathogenesis; studying allele-specific diabetogenicity (e.g., A2 vs. B39); testing antigen-specific tolerance strategies; discovery of autoantigenic epitopes.	Strong and reproducible T1D incidence; allows allele-specific modeling; lack of mouse MHC-I provides cleaner background.	β2m deficiency causes loss of FcRn, thus limiting testing antibody- or serum-albumin-based T1D interventions (limitation addressed in NOD.cMHCI^-/-^ mice); restricted HLA-I diversity and absence of HLA-II.	[140,141,142]
Humanized HLA.DQ8-Tg NSG mice	NSG mice expressing human HLA-DQ8 and humanized with HLA-DQ8^+^ human fetal thymus and CD34^+^ HSCs; models HLA-DQ8-restricted human T-cell responses to β-cell antigens. **Disease course and time frame:** Induced human T-cell-mediated diabetes; requires beta-cell stress and antigenic challenge; onset over weeks. **Genetic background:** HLA.DQ8–Tg NOD.SCID x NSG.	Human CD4^+^ T cells from ‘donor’ humanized mice engineered for expression of insulin-B:9–23-specific TCRs (HLA-DQ8/8 patient-derived) cause insulitis and diabetes upon adoptive transfer into streptozotocin-induced ‘recipients’ (humanized mice immunized with insulin-B:9–23).	Investigation of diabetogenic epitopes and pathogenic TCRs; antigen-specific immunotherapy; preclinical testing.	HIS allows for better post-immunization responses in secondary lymphoid organs; Antigen-specific, HLA-restricted human T-cell–driven diabetes.	Requires β-cell stress (not spontaneous); CD4^+^ T-cell-centric with limited CD8^+^ T contribution.	[143]
YES and YES-RIP-hB7.1 mice	NOD mice lacking mINS and MHC-I/II but expressing HLA-A*02:01, HLA-DQ8, and hINS transgenes (YES). **Disease course and time frame:** No spontaneous diabetes for up to 1 year. **Genetic background:** NOD/ShiLtJ with cMHC-I/II and mINS deficiency YES-RIP-hB7.1 mice express human costimulatory molecule B7.1 (hB7.1) in pancreatic β cells on the YES background. **Disease course and time frame:** Spontaneous/accelerated diabetes due to beta-cell costimulation. **Genetic background:** NOD/ShiLtJ with cMHC-I/II and mINS deficiency.	No spontaneous diabetes for up to 1 year; T1D induced with polyinosinic-polycytidylic acid (poly(I:C)). YES-RIP-hB7.1 allows development of spontaneous diabetes via local costimulation within the islets.	Evaluate immune responses to human insulin, studying phases of disease pathogenesis. Discovery of new pathogenic epitopes in human insulin; study role of costimulation in disease pathogenesis.	High translational relevance due to HLAs and auto-antigen; models both CD4^+^ and CD8^+^ T responses. Addresses shortcomings of YES mice for spontaneous disease.	No spontaneous diabetes; poly(I:C) trigger is artificial; β-cell environment is still murine. Moderate insulitis compared to NOD.	[144,145]

**Table 4:** Animal models for rheumatoid arthritis.

Model	Description	Phenotype and Pathology	Applications	Advantages	Limitations	References
***Murine Models***						
Collagen-induced Arthritis (CIA)	Immunization of genetically susceptible mice (e.g., DBA/1 expressing *H-2^q^* haplotype of murine *I-A*) with type-II heterologous collagen (CII) in complete Freund’s adjuvant (CFA). **Disease course and time frame**: Acute-onset polyarthritis ~21–35 days after CII immunization; may become chronic/relapsing. **Genetic background:** Most commonly DBA/1 with H-2q haplotype; C57BL/6 can be used with modified protocols.	Disease manifests 21–25 days after immunization; symmetrical inflammatory polyarthritis; synovial hyperplasia; leukocyte infiltration; complement activation; pannus formation; high anti-CII antibodies and collagen-specific T cells; systemic TNF, IL-6, IL-1β, IL-17.	Studying antigen-specific T- and B-cell responses and FcγR/complement effector pathways; preclinical testing of biologics/anti-rheumatic drugs (Anti-TNF/IL6R Abs, JAK inhibitors and anti-RANKL); role of microbiota in RA.	Robust and reproducible—gold standard for RA; mimics HLA-II susceptibility in humans; B and T-cell-mediated pathology; numerous inbred mouse strains available to study contributing genetic and microbial factors.	Requires induction and has variable onset; induction varies with mouse strain/age, CII source and emulsion technique.	[167,168,169,170,171,172]
Collagen-antibody induced arthritis (CAIA)	Passive transfer of anti–CII monoclonal Abs and lipopolysaccharide (LPS) in susceptible mice; T/B-cell-independent but progression is enhanced by CII-reactive T cells. **Disease course and time frame**: Acute effector-phase arthritis; onset ~24–72 h after antibody transfer; usually resolves within ~2–3 weeks. **Genetic background:** BALB/c, DBA/1, C57BL/6, or other recipient strains depending on antibody/LPS protocol.	T- and B-cell independent arthritis; Rapid onset of synovitis with infiltration of macrophage and neutrophils and Fc-activated complement; vascular opacification, cartilage degradation and bone erosion.	Immune complex-mediated effector mechanisms; FcγR and complement biology; innate cytokine pathways; rapid preclinical drug testing.	Synchronous disease onset, highly reproducible; allows use of congenic, transgenic, and knockout mice; bypasses adaptive priming; commercially available cocktail of monoclonal antibodies allows ease of use.	Rapid onset; acute disease; no antigen-specific T/B responses; collagen-focused autoimmunity; limited modeling of preclinical RA.	[173,174,175,176]
Proteoglycan- induced Arthritis (PGIA)	Repeated immunization with human cartilage proteoglycan in adjuvant in BALB/c (H-2^d^) and C3H mice **Disease course and time frame:** Chronic progressive arthritis; onset after repeated immunizations, usually ~7–10 weeks. **Genetic background:** BALB/c, especially H-2d, and C3H backgrounds; strongly MHC-II restricted.	Late-onset, chronic, symmetrical polyarthritis, pannus formation and synovial infiltration; cartilage loss and bone erosion; high PG antibody titers causing synovial macrophage and fibroblast activation; Th1/Th17 cytokine profile-choice of adjuvant determines the cytokine profile; only model affecting the axial skeleton.	Antigen-specific T- and B-cell responses; autoantibody generation; evaluating cartilage-protective drugs; cytokine network studies.	Chronic progressive course resembling human RA progression; strong adaptive immunity; robust autoantibody response; models relapsing disease; only model affecting the axial skeleton.	Requires multiple immunizations; slower and variable onset; strain restricted; heterologous antigen-driven; limited ACPA relevance.	[177,178,179]
Antigen- induced Arthritis (AIA)	Induced by intra-articular injection of antigen (methylated bovine serum albumin in CFA) into knee joints of pre-immunized mice **Disease course and time frame:** Acute monoarthritis, onset within days after intra-articular antigen injection; chronic disease with repeated antigen challenge. **Genetic background:** C57BL/6, BALB/c, or C3H backgrounds.	Immune responses against cartilage’s C-I/II and proteoglycans; initial acute immune complex-mediated inflammation followed by T-cell-mediated chronic synovial hyperplasia and subsequent bone destruction of injected joint (monoarthritis); progression is dependent on IL-23/IL-17Ra and Treg depletion.	Studying antigen-specific T-cell-mediated joint inflammation; role of Tregs and costimulatory pathways; evaluating cartilage and bone anti-erosive therapies; study mechanisms of pain and bone loss.	Highly reproducible; synchronized disease onset; monoarthritis allows internal contralateral joint control; no strain restrictions; strong antigen specificity; ideal for mechanistic T-cell studies.	Non-spontaneous; minimal B-cell involvement (no antibodies); chronic disease modeling requires repeated antigen challenge.	[180,181,182,183,184,185]
K/BxN mice and K/BxN serum transfer arthritis	K/BxN mice express KRN Tg-TCR (reactive to glucose-6-phosphate isomerase (GPI) peptide 282–294) presented by the NOD-derived I-A^g7^. K/BxN serum transfer model: transfer of serum from K/BxN mice reliably causes arthritis in a wide range of recipient strains. **Disease course and time frame:** K/BxN: spontaneous arthritis from ~3–4 weeks; serum-transfer model: acute arthritis within ~24–48 h. **Genetic background:** KRN TCR Tg on C57BL/6 x I-A^g7^-expressing NOD background; serum transfer works in many recipient strains.	Severe spontaneous inflammatory arthritis; activated KRN^+^ T cells help B cells to generate anti-GPI antibodies, causing rapid and chronic symmetric synovitis. Anti-GPI antibodies mediate FcγR/C5 complement activation, neutrophil/macrophage recruitment, and IL-1β and TNF-driven inflammation; transfer of serum with anti-GPI antibodies causes transient, self-resolving arthritis (serum-transfer model).	Mechanisms of autoantibody-induced arthritis; effector-phase RA studies involving FcγR biology and complement pathways; innate cell contributions; testing cytokine blockade and anti-inflammatory therapies.	Highly reproducible; robust and synchronized onset; serum-transfer system allows assessment of genetic determinants of effector phase independent of T-cell tolerance-breaking triggers.	Antigenic specificity in K/BxN mice does not fully mirror human RA; serum transfer model exhibits limited chronicity (repeated serum transfer required) and lacks T-cell-dependent priming phase.	[186,187,188,189,190]
SKG mice	BALB/c mice with a spontaneous hypomorphic mutation in TCR signal transduction protein ZAP-70 (causing autoreactive CD4^+^ T cells to escape thymic negative selection). **Disease course and time frame:** Chronic; develops over weeks after environmental trigger such as zymosan/beta-glucan. **Genetic background:** BALB/c background carrying hypomorphic Zap70 mutation.	Spontaneous chronic polyarthritis upon environmental trigger (e.g., fungal β-glucans) activates innate dectin-1 signaling and complement pathway, causing expansion of Th17 cells; elevated IL-17, IL-6, TNF-α and IL-1β; synovial hyperplasia and bone erosion; RF production.	Study defects in thymic selection; Th17-driven autoimmunity; gene-environment interactions; innate immune triggers of autoimmune arthritis; evaluating RA therapies in early disease.	Genetic model of defective thymic selection; strong T-cell-driven pathology; recapitulates chronic polyarthritis like RA; demonstrates interaction between genetic susceptibility and environmental triggers.	Requires environmental trigger; mutation uncommon in human RA; Th17-dominant pathology does not reflect full RA heterogeneity; female gender bias.	[191,192,193,194]
Human TNF-Tg mice	Genetically engineered mice overexpressing deregulated (over-stabilized) expression of human TNF-α **Disease course and time frame:** Chronic spontaneous inflammatory arthritis; onset ~3–6 weeks; progressive. **Genetic background:** C57BL/6 or mixed backgrounds.	Spontaneous chronic arthritis beginning at ~4–6 w of age. TNF-driven inflammation with macrophage and neutrophil infiltration; increased IL-1 and IL-6; synovial hyperplasia; cartilage destruction and bone resorption	Studying TNF-driven pathogenesis of RA and testing anti-TNF or anti-cytokine therapies	Spontaneous and stable disease; robust and reproducible; strong resemblance to TNF-mediated inflammation in RA; historically validated therapeutic targets	Artificial TNF-driven; lacks autoimmune initiation mechanisms; minimal autoantibody involvement; may overemphasize TNF-dependent mechanisms	[195,196,197,198]
IL-1R antagonist KO (*Il1rn*^−/−^) mice	BALB/c mice with genetic deletion of IL-1R antagonist (that competes with IL-1 for binding to type I IL-1 receptors) resulting in uncontrolled IL-1 signaling. **Disease course and time frame:** Chronic spontaneous arthritis; onset ~5–8 weeks. **Genetic background:** BALB/c is highly susceptible, C57BL/6 is highly resistant.	Spontaneous chronic arthritis ~5–8 w of age; Elevated IL-1 signaling; expansion of Th17 cells; activation of IL-17-producing CCR2^+^Vγ6^+^ γδ T cells in inflamed joints; increased TNF-α, IL-6, and IL-17; synovial hyperplasia, bone and cartilage damage; autoantibody production.	Studies of IL-1 signaling and Th17-driven inflammation; cytokine network interactions, testing anti-IL-1 or anti-IL-17 therapies.	Spontaneous autoimmune arthritis; strong inflammatory phenotype; involves both innate and adaptive immunity.	Artificial cytokine dysregulation; strain-dependent severity; lacks autoimmune initiation mechanisms.	[199,200,201,202]
***Humanized mouse models***						
**(a) HLA-DR Tg or TCR Tg mice**						
HLA-DR Tg mice (DR1/DR4/DR4.CD4)	HLA-Tg mice expressing RA-associated risk alleles HLA-DRA1*0101 or DRB1*0401 allowing presentation of RA-relevant autoantigens (e.g., type-II collagen [CII]); endogenous murine I-E expression is often retained; some models also express transgenic human CD4 in T cells (DR4.CD4 mice). **Disease course and time frame:** CII-induced arthritis; onset ~2–4 weeks. **Genetic background:** C57BL/6 or mixed backgrounds.	HLA-DR4–restricted CD4^+^ T-cell responses to CII (position 259–273); produce anti-CII antibodies; inflammatory polyarthritis with synovitis and joint destruction upon CII immunization.	Study HLA-DR-restricted peptide presentation/responses and influence of RA risk alleles; validation of immunodominant synovial autoantigen epitopes (such as CII, HCgp-39, proteoglycan aggrecan, fibrinogen, and vimentin); modeling gene–environment interactions (e.g., smoking, microbiome).	Strong RA genetic relevance; robust antigen-specific CD4 responses.	Not spontaneous; murine immune system present; citrullinated proteins rather than CII are the dominant antigen in human RA.	[203,204,205]
HLA-DRB1*0401.AE(o) mice	Mice lacking all 4 murine MHC-II (Aα, Aβ, Eα, and Eβ), with transgenic expression of HLA- DRB1*0401, an RA risk allele. **Disease course and time frame:** CII-induced arthritis; onset ~2–4 weeks; reproduces female sex bias. **Genetic background:** C57BL/6-derived AEo/MHC-II-deficient background.	Increased disease susceptibility in females upon CII immunization; production of Th1 cytokines (IFNγ, IL-18, and TNFα) and higher splenic cellularity (CD3^+^ and CD4^+^) in females than males; production of RF and anti-CCP (anti-cyclic citrullinated peptide) antibodies; inflammatory joint damage.	Understanding sex bias in RA pathogenesis; role of female hormones; B cells and ‘shared-epitope’ mechanisms; evaluating peptide-based tolerance strategies and antigen-specific immunotherapies.	Reproducible collagen-induced arthritis; mimics sex bias as seen in human RA.	Not spontaneous; murine immune system despite human HLA expression; citrullinated proteins rather than CII is dominant antigen in RA; does not fully capture chronic heterogeneity of RA.	[206,207]
HLA-DQ8. AE(o) mice	Mice lacking murine MHC-II with HLA-DQ8 (encoded by DQA1*0301/ DQB1*0302). **Disease course and time frame:** CII-induced arthritis; onset ~2–4 weeks. Increased clinical manifestation when co-expressed with RA risk allele DRB1*0401. **Genetic background:** C57BL/6-derived AEo/MHC-II-deficient background.	CD4^+^ T-cell-mediated disease; immunization with CII induces severe CIA with strong autoreactive T- and B-cell responses. In humans, HLA-DQB1*03 occurs in linkage with the RA risk allele HLA-DRB1*0401 (while HLA-DRB1*0402 is relatively protective)—enhanced disease in DR4*0401.DQ8 double-Tg mice than DR4*0402.DQ8 double-Tg mice.	Studying HLA class-II-mediated RA susceptibility; modeling gene–environment interactions (e.g., smoking, microbiome); evaluating antigen-specific immune responses to citrullinated proteins; preclinical testing of immuno-modulatory therapies targeting CD4^+^ T cells or B cells.	Closely mimics HLA-associated genetic risks; study of antigen-specific CD4^+^ T-cell responses; delineates gene complementation between DR and DQ in RA; captures genetic-environmental factors; intra-tracheal bleomycin injection allows study of RA-associated lung pathology.	Not spontaneous; murine immune system despite human HLA expression; citrullinated proteins rather than CII is dominant antigen in RA; does not fully capture chronic heterogeneity of human RA.	[208,209,210,211,212]
HLA-DR4 + autoantigen Tg mice	Double transgenic mice expressing RA-associated HLA-DRB1*0401 along with autoantigens like human collagen, immuno-dominant T-cell epitope within mouse CII (MMC), or citrullinated proteins. **Disease course and time frame:** Induced/antigen-driven arthritis depending on autoantigen and immunization protocol. **Genetic background:** C57BL/6-derived HLA-DR4 Tg x human autoantigen Tg.	Arthritis with synovitis, cartilage destruction, bone erosion; anti-collagen or anti-citrullinated protein responses (ACPA).	Study of HLA-restricted T- and B-cell responses to human autoantigens; RA pathogenesis; epitope mapping and antigen presentation studies; mechanisms of citrullination-driven autoimmunity.	Direct modeling of human HLA risk allele + relevant autoantigen; enables identification of arthritogenic peptides; highly relevant to human RA.	Collagen is not a dominant human RA autoantigen compared with citrullinated proteins; the model relies on a murine immune context, does not fully recapitulate chronic progressive RA, and often requires supra-physiologic autoantigen exposure.	[210,213,214,215]
TCR Tg mice	Mice expressing Tg TCRαβ specific for an arthritogenic epitope of human autoantigen (e.g., CII; proteoglycan (aggrecan) or human cartilage glycoprotein (HCgp-39). **Disease course and time frame:** Antigen-specific arthritis; induced or spontaneous depending on TCR specificity and trigger. **Genetic background:** DBA/1 or B10.Q/H-2q for CII-specific TCR models; BALB/c for proteoglycan/ aggrecan-specific systems.	Antigen-specific CD4^+^ T-cell-driven arthritis characterized by activation and expansion of autoreactive T cells, synovial leukocyte infiltration, synovial hyperplasia, pannus-like tissue formation, cartilage damage, and bone erosion; disease severity and joint pathology depend on the target autoantigen and usually require antigenic challenge.	Identification of arthritogenic epitopes; role of antigen-specific T cells in arthritis induction; mechanisms of autoantigen-specific T-cell activation; tolerance studies.	Use of well-established RA antigens; reproducible responses; useful for CIA or PGIA mechanistic dissection.	Requires immunization/adjuvant; collagen not dominant antigen in human RA; limited antigen diversity; mouse immune context.	[213,216,217]
HLA-DR1 + TCR Tg mice	Double-Tg mice expressing HLA-DRB1*0101 (haplotype susceptible to CIA) and autoreactive CII-specific TCR^+^ CD4 T cells. **Disease course and time frame:** Antigen-specific arthritis; accelerated disease in double-transgenic systems. **Genetic background:** C57BL/6-derived HLA-DR1 Tg x CII-specific TCR Tg.	Accelerated and more severe form of CIA than their DR1 Tg littermates following immunization with bovine CII/CFA; CD62L^low^CD44^High^ activated T cells expressing Th1/Th17 cytokines; modified collagen peptide (A12) induces tolerance.	Testing novel therapeutic approaches; immune tolerance strategies.	Human-relevant HLA-restricted antigen presentation; reproducible and mechanistically clean system; ideal for tracking antigen-specific responses.	Artificially high TCR frequency; requires immunization; limited antigen diversity (single specificity); murine immune system.	[218]
***(b) Human RA tissue/immunodeficient mice chimeras***						
RA synovium/ SCID mouse chimera	Human RA synovial tissue implanted (under renal capsule or joints) into SCID mice; inflammatory stimuli required to retain immune cells in graft. Optional co-implantation of normal human cartilage. **Disease course and time frame:** Short-lived graft model; inflammation assessed over ~2–6 weeks after implantation and declines within weeks unless sustained by stimuli. **Genetic background:** SCID.	Synovial pannus formation, cartilage infiltration by fibroblast-like cells, production of human cytokines (TNF, IL-6), matrix degradation; destruction of co-implanted normal cartilage (if performed).	Study RA synovial pathology, effect of inflammatory cells/mediators/inhibitors on angiogenesis; mechanisms of anti-rheumatic drugs; testing biologics (anti-TNF and anti-IL-6), and T/B-cell-related therapies (anti-CTLA4/CD20/IL-17 antibodies).	Direct modeling of human RA synovial microenvironment; preserves patient-specific pathology, both human and murine granulocytes contribute to inflammation.	No systemic autoimmunity; limited immune-cell diversity; short-lived graft behavior (inflammatory stimuli always required).	[219,220,221,222,223]
RA-Synovial Fibroblast/SCID mouse chimera	SCID mice co-implanted with synovial fibroblasts from RA patients (RA-SF) and normal cartilage in a gel sponge (substitutes the synovial matrix as a carrier for synovial fibroblasts to avoid bias from cellular and matrix components). **Disease course and time frame:** Progressive local cartilage invasion model; cartilage attachment/invasion is typically assessed over ~4–8 weeks after co-implantation. **Genetic background:** SCID.	Cartilage destruction solely mediated by invasive RA-SF independent of T cells/macrophages/inflammation; model mimics progressive joint involvement (oligo- to polyarticular spread) driven by fibroblast behavior.	Study of fibroblast-mediated cartilage invasion and destruction in RA; investigate intrinsic pathogenic properties of RA-SF; evaluate therapies targeting invasiveness and tissue degradation; dissect non-immune, stromal-driven mechanisms of joint damage.	Provides a controlled, non-inflammatory environment isolating fibroblast-specific effects; useful for studying direct tissue invasion mechanisms; use of primary human RA-SF provides clinical relevance.	No T/B-cell responses; immune-cell context lacking; does not model antigen-specific processes or disease initiation and progression; represents only stromal aspect of RA pathology.	[219,224,225]
RA-Synovial fluid mononuclear cells/SCID chimera	SCID mice injected (intra-articularly/intra-peritoneally) with in vitro stimulated RA-SMC (mononuclear cells (mainly T cells) isolated from RA synovial fluid/tissue. **Disease course and time frame:** Progressive local cartilage invasion model; cartilage attachment/invasion is typically assessed over ~4–8 weeks after co-implantation. **Genetic background:** SCID.	Synovial hyperplasia and polyarthritis; production of autoantibodies; T-cell-driven disease with oligoclonal expansion of pathogenic T cells.	Study pathogenic T-cell clones and local antigen-driven immune responses; investigate TCR repertoire and clonality; test therapies targeting T-cell-mediated synovial inflammation.	Direct use of disease-relevant human synovial immune cells; captures joint-specific immune responses and antigen-driven T-cell expansion.	Disease induced only upon in vitro T-cell stimulation; limited systemic features of RA.	[226,227]
Hu-HSC in NOG or NSG chimera	NSG/NOG mice transplanted with human CD34^+^ HSCs from cord blood or bone marrow (optional injection with CFA or EBV as stimuli). **Disease course and time frame:** Trigger-dependent; arthritis develops over weeks to months after human CD34^+^ HSC engraftment, usually following CFA or EBV stimulation. **Genetic background:** NOG or NSG.	Erosive arthritis with pannus formation, bone marrow edema, synovial hyperplasia (mainly T-cell infiltration) in both models; about 65% of low dose EBV-infected humanized NOG mice showed disease; RF and ACPA not seen.	Study possible role of viral infection (e.g., EBV), in triggering RA-like pathology. T-cell-mediated joint inflammation with engrafted human leukocytes; evaluate anti-inflammatory therapies (e.g., TNF blockade).	Complete humanized system with generation of all major immune subsets; reproduces key pathology in human RA.	Stimulation/pathologic trigger required; does not reproduce serologic RA features (i.e., RF or ACPA); model does not use RA-patient HSCs.	[228,229]
NSG-RA mice	NSG mice reconstituted with PBMCs from RA patients, followed by arthritis exacerbation using anti-CII antibody cocktail and LPS. **Disease course and time frame:** Acute-phase arthritis; requires anti-CII antibody cocktail and LPS; develops over ~1–2 weeks. **Genetic background:** NSG.	RA-like inflammatory arthritis with paw swelling, synovitis, pannus formation, proteoglycan loss, bone erosion, infiltration of human CD4^+^ T cells, CD8^+^ T cells, CD14^+^ monocytes and CD19^+^ B cells; increased human inflammatory cytokines including IFNγ, TNFα, IL-12p70 and IL-17A.	Study patient-derived immune-cell contributions to RA-like synovitis; investigate human leukocyte–synovial fibroblast interactions, Th1/Th17 pathways, autoantibody responses, and therapeutic responses to anti-inflammatory biologics.	Uses easily accessible patient PBMCs; preserves donor immune-cell heterogeneity; reproduces key RA-like histopathological features; responds to prednisolone and infliximab treatment.	Acute-phase model; requires anti-CII antibody/LPS challenge; murine stromal and joint tissue; does not fully model chronic RA initiation or long-term progressive joint destruction.	[230]

## Data Availability

No new data were collected for this review.

## Abbreviations

The following abbreviations are used in this manuscript:

ACPA	Anti-citrullinated protein antibodies
APC	Antigen-presenting cell
BBB	Blood–brain barrier
BLC	B lymphocyte chemoattractant
CAIA	Collagen antibody-induced arthritis
CCP	Cyclic citrullinated peptide
CCR2	C-C chemokine receptor type 2
CCR6	C-C chemokine receptor type 6
CXCL	C-X-C motif chemokine ligand
CD	Crohn’s disease
CD3/CD4/CD8/CD20/CD25/CD34/CD45/CD68/CD147	Cluster of differentiation surface markers (lymphocyte/leukocyte subsets)
CIA	Collagen-induced arthritis
CII (C-II)	Type-II collagen
CNS	Central nervous system
CT	Computed tomography
CTLA-4	Cytotoxic T lymphocyte–associated antigen 4
DAI	Disease Activity Index
DC	Dendritic cell
DSS	Dextran sulfate sodium
EAE	Experimental autoimmune encephalomyelitis
EBV	Epstein–Barr virus
Foxp3	Forkhead box P3
GAD65	Glutamic acid decarboxylase 65
GM-CSF	Granulocyte–macrophage colony-stimulating factor
GWAS	Genome-wide association studies
HIS	Human immune system (mice)
HLA	Human leukocyte antigen
HLA-DR/DR1/DR2/DR4/DR15/DRB1/DRB5/DQ8/DQB1	HLA class-II alleles (haplotypes referenced in autoimmune susceptibility)
HSC	Hematopoietic stem cell
IA-2	Insulinoma-associated antigen-2
IAA	Insulin autoantibodies
IBD	Inflammatory bowel disease
IFN-γ	Interferon-gamma
IKK-β	Inhibitor of nuclear factor-κB kinase subunit beta
IL-1α/IL-1β/IL-2/IL-6/IL-10/IL-13/IL-17/IL-17A/IL-23	Interleukin (cytokine family members)
IL-23R	Interleukin-23 receptor
IL2RA	Interleukin-2 receptor alpha chain (CD25)
INS	Insulin
IP-10	Interferon Gamma-Induced Protein 10
KC	Keratinocyte-derived Chemokine
MIP-3α, MIP-1α	Macrophage Inflammatory Proteins
MBP	Myelin basic protein
MCP-1	Monocyte Chemoattractant Protein-1
M-CSF	Macrophage colony-stimulating factor
MHC	Major histocompatibility complex
MIG	Monokine Induced by Gamma Interferon
MOG	Myelin oligodendrocyte glycoprotein
MS	Multiple sclerosis
MUC2	Mucin 2
NEMO	NF-κB essential modulator
NF-κB	Nuclear factor kappa-light-chain-enhancer of activated B cells
NK-T cell	Natural killer T cell
NOD	Non-obese diabetic
NOD2	Nucleotide-binding oligomerization domain-containing protein 2
NSG	NOD-scid IL2Rγ-null
NSG-UC	NSG-based ulcerative colitis humanized mouse model
PADI4	Peptidyl arginine deiminase 4
PBMC	Peripheral blood mononuclear cell
PLP	Proteolipid protein
PTPN22	Protein tyrosine phosphatase non-receptor type 22
RA	Rheumatoid arthritis
RAG-2	Recombination activating gene 2
RANKL	Receptor activator of nuclear factor-κB ligand
RANTES	Regulated upon Activation, Normal T-cell Expressed and Secreted
RF	Rheumatoid factor
RIP	Receptor-interacting protein (kinase)
ROS	Reactive oxygen species
SCID	Severe combined immunodeficiency
SJL	Swiss Jim Lambert (mouse strain)
SMASH	Standardized Microscopic Arthritis Scoring of Histological sections
STAT4	Signal transducer and activator of transcription 4
STAT5	Signal transducer and activator of transcription 5
T1D	Type-1 diabetes
TCR	T-cell receptor
Tg	Transgenic
Th1/Th2/Th17	T helper cell subsets (type 1, type 2, type 17)
TNBS	2,4,6-Trinitrobenzenesulfonic acid
TNF-α	Tumor necrosis factor-alpha
TRAF1	TNF receptor-associated factor 1
TRANCE	TNF-related activation-induced cytokine (RANKL)
Treg	Regulatory T cell
UC	Ulcerative colitis
ZAP-70	Zeta-chain–associated protein kinase 70
ZnT8	Zinc transporter 8
